# MR-Clust: clustering of genetic variants in Mendelian randomization with similar causal estimates

**DOI:** 10.1093/bioinformatics/btaa778

**Published:** 2020-09-11

**Authors:** Christopher N Foley, Amy M Mason, Paul D W Kirk, Stephen Burgess

**Affiliations:** MRC Biostatistics Unit, School of Clinical Medicine, University of Cambridge, Cambridge CB2 0SR, UK; Department of Public Health and Primary Care, Cardiovascular Epidemiology Unit, University of Cambridge, Cambridge CB1 8RN, UK; MRC Biostatistics Unit, School of Clinical Medicine, University of Cambridge, Cambridge CB2 0SR, UK; Cambridge Institute of Therapeutic Immunology & Infectious Disease, University of Cambridge, Cambridge CB2 0AW, UK; MRC Biostatistics Unit, School of Clinical Medicine, University of Cambridge, Cambridge CB2 0SR, UK; Department of Public Health and Primary Care, Cardiovascular Epidemiology Unit, University of Cambridge, Cambridge CB1 8RN, UK

## Abstract

**Motivation:**

Mendelian randomization is an epidemiological technique that uses genetic variants as instrumental variables to estimate the causal effect of a risk factor on an outcome. We consider a scenario in which causal estimates based on each variant in turn differ more strongly than expected by chance alone, but the variants can be divided into distinct clusters, such that all variants in the cluster have similar causal estimates. This scenario is likely to occur when there are several distinct causal mechanisms by which a risk factor influences an outcome with different magnitudes of causal effect. We have developed an algorithm MR-Clust that finds such clusters of variants, and so can identify variants that reflect distinct causal mechanisms. Two features of our clustering algorithm are that it accounts for differential uncertainty in the causal estimates, and it includes ‘null’ and ‘junk’ clusters, to provide protection against the detection of spurious clusters.

**Results:**

Our algorithm correctly detected the number of clusters in a simulation analysis, outperforming methods that either do not account for uncertainty or do not include null and junk clusters. In an applied example considering the effect of blood pressure on coronary artery disease risk, the method detected four clusters of genetic variants. A *post hoc* hypothesis-generating search suggested that variants in the cluster with a negative effect of blood pressure on coronary artery disease risk were more strongly related to trunk fat percentage and other adiposity measures than variants not in this cluster.

**Availability and implementation:**

MR-Clust can be downloaded from https://github.com/cnfoley/mrclust.

**Supplementary information:**

Supplementary data are available at *Bioinformatics* online.

## 1 Introduction

Genome-wide association studies have discovered many genetic variants associated with various traits and conditions. Such genetic variants can aid understanding of the biological mechanisms that influence traits (Plenge *et al.*, 2013). They can also be used to link modifiable traits to disease outcomes. Due to random mating (i.e. the choice of partner is independent of the genetic variants under investigation) and Mendel’s laws of segregation and independent assortment, genetic variants are typically distributed independently of traits that they do not directly influence, and so can be treated similarly to random treatment assignment in a randomized controlled trial (Davey Smith and Ebrahim, 2003; Lawlor *et al.*, 2008). Genetic variants associated with a given trait are therefore plausible instrumental variables (IVs) for that trait (Didelez and Sheehan, 2007). The use of genetic variants as IVs to assess the causal effect of a risk factor on an outcome is known as Mendelian randomization (Burgess and Thompson, 2015).

While the hypothesis of whether a risk factor has a causal effect on an outcome can be assessed with a single valid IV (Didelez and Sheehan, 2007), most genetic variants do not explain enough variability in the risk factor to have sufficient power to reliably detect a moderate-sized causal effect. Additionally, it is prudent to use all relevant data to address the causal hypothesis of interest. Under strict parametric assumptions (described below), the causal estimates based on each valid IV will target the same causal parameter—the average causal effect (Hernán and Robins, 2006). Excess heterogeneity between causal estimates from different genetic variants is often interpreted as evidence that not all genetic variants are valid IVs (Verbanck *et al.*, 2018).

However, it may be that different genetic variants influence the risk factor in distinct ways, leading to heterogeneity between causal estimates calculated using different variants. For example, several hundred genetic variants have been demonstrated to be independently associated with blood pressure (Evangelou *et al.*, 2018). Different genetic variants may influence blood pressure via distinct biological mechanisms. Alternatively, some variants may have pleiotropic effects on traits that are causally upstream of blood pressure rather than blood pressure directly. Or it may be that blood pressure is in fact a composite trait consisting of multiple components that is captured only as a single measurement. Variants that influence the risk factor in a similar way are likely to have similar causal estimates.

Several previous attempts have been made to cluster genetic variants that are associated with a given risk factor. Walter *et al.* (2015) took 32 genetic variants associated with body mass index (BMI) and divided the variants into four groups based on biological understanding of the function of the variants. They then compared the causal estimates of BMI on depression based on each group of variants. Udler *et al.* (2018) took 94 variants associated with Type 2 diabetes, and divided the variants into 7 groups based on their associations with 47 diabetes-related traits. Tanigawa *et al.* (2019) applied a truncated singular value decomposition method to genetic association estimates from the UK Biobank study to find clusters of variants having similar associations with a range of traits.

In this article, we introduce a method to cluster variants that have similar causal estimates for the given risk factor and outcome. As we do not use data on genetic associations with alternative traits to form the clusters, an advantage of this approach is that genetic associations with traits can be used to validate the division into clusters. If traits can be found that predict cluster membership, this increases the plausibility that the clusters have a biological interpretation. We refer to our method as MR-Clust.

Our manuscript is structured as follows. First, we provide an overview of Mendelian randomization, and introduce the modelling assumptions and notation used in Section 2. We also consider factors that may lead to heterogeneity between causal estimates based on different genetic variants, and in particular investigate how this would lead to clustered heterogeneity. Next, we introduce a statistical approach for detecting clusters of variants with similar causal estimates, which are likely to influence the risk factor in a similar way (Section 3). There are two distinct aspects of our method over conventional applications of clustering. First, we account for differential uncertainty in the causal estimates that we are clustering. Second, we include a ‘junk’ cluster in our model, so that variants with estimates that do not fit into any clusters are included in the junk cluster rather than any other cluster. We apply our method in a simulation study, and to consider 180 independent genetic variants associated with blood pressure at a genome-wide level of significance, and find clusters in the causal estimates of blood pressure traits on coronary artery disease (CAD) risk (Section 4). We conclude by discussing the results of the manuscript, and their application to epidemiological practice (Section 5).

## 2 Materials and methods

The aim of a Mendelian randomization analysis is to establish whether there exists a causal relationship between a risk factor *X* and an outcome *Y* using genetic variants *G_j_*, j=1,2,…,J as IVs. An additional aim is to estimate the causal effect of the risk factor on the outcome. In this section, we introduce assumptions and methods for IV estimation, and discuss when the estimates based on different IVs will be similar and when they will be different.

### 2.1 Instrumental variable assumptions

A genetic variant *G_j_* is a valid IV if it satisfies three assumptions:

- (relevance) it is associated with the risk factor,
- (exchangeability) its association with the outcome is not confounded and
- (exclusion restriction) it has no effect on the outcome except that mediated via the risk factor (Clarke and Windmeijer, 2012; Greenland, 2000).

Under these assumptions, any association between the genetic variant and the outcome is indicative of a causal effect of the risk factor on the outcome (Baiocchi *et al.*, 2014).

To estimate a causal parameter, we make further parametric assumptions of linearity and homogeneity in the relationships between the genetic variant, risk factor and outcome. Specifically:

(1) $$E(X|G_{j}=g)=\beta_{Xj0}+\beta_{Xj}\;g,$$

 

(2) $$E(Y|G_{j}=g)=\beta_{Yj0}+\beta_{Yj}\;g,$$

**Fig. 1.**
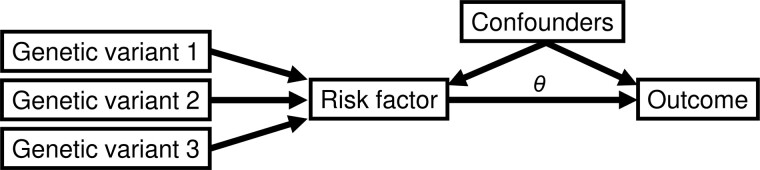
Directed acyclic graph illustrating relationships between three genetic variants that are valid IVs with a risk factor, outcome and confounders of the risk factor–outcome associations. The causal effect of the risk factor on the outcome is indicated by *θ*

 

(3) $$E(Y|\text{do}(X=x))=\theta_{0}+\theta \;x,$$

where *θ* is the average causal effect of the risk factor on the outcome (Angrist *et al.*, 1996), and do(X=x) is Pearl’s do operator, meaning that the risk factor is intervened on to take value *x* (Pearl, 2000). This model can be illustrated as a directed acyclic graph (Fig. 1).

It can be shown that *θ* can be estimated consistently as the ratio of the estimated genetic association with the outcome divided by the estimated genetic association with the risk factor (Didelez *et al.*, 2010):

(4) $${\hat{\theta }}_{j}=\frac{{\hat{\beta }}_{Yj}}{{\hat{\beta }}_{Xj}},$$

which we call the ratio estimate of the *j*th variant. The standard error of this quantity ${\hat{\sigma }}_{j}=\text{se}({\hat{\theta }}_{j})$ can be estimated using the delta method:

(5) $${\hat{\sigma }}_{j}=\left|\frac{\text{se}({\hat{\beta }}_{Yj})}{{\hat{\beta }}_{Xj}}\right|\;(\text{first}\;\text{order})\;\text{or}$$

 

(6) $${\hat{\sigma }}_{j}=\sqrt{\frac{\text{se}{\left({\hat{\beta }}_{Yj}\right)}^{2}}{{\hat{\beta }}_{Xj}^{2}}+\frac{{\hat{\beta }}_{Yj}^{2}\;\text{se}{\left({\hat{\beta }}_{Xj}\right)}^{2}}{{\hat{\beta }}_{Xj}^{4}}-\frac{2\rho \;{\hat{\beta }}_{Yj}\;\text{se}({\hat{\beta }}_{Yj})\;\text{se}({\hat{\beta }}_{Xj})}{{\hat{\beta }}_{Xj}^{3}}}\;(\text{second}\;\text{order}),$$

where *ρ* is the correlation between the genetic association estimates ${\hat{\beta }}_{Yj}$ and ${\hat{\beta }}_{Xj}$. This parameter cannot be estimated directly from summarized data, but it will be zero in a two-sample setting (i.e. the genetic associations with risk factor and outcome are estimated in non-overlapping datasets). When there is sample overlap, it can be specified by the user based on the degree of overlap and the expected correlation between the risk factor and outcome (Burgess *et al.*, 2016a). If second-order weights are used, then a sensitivity analysis for this parameter is advised.

We note that these parametric assumptions are sufficient, but not necessary for the estimation of the average causal effect; weaker assumptions have been proposed (Swanson and Hernán, 2013). Alternatively, under the monotonicity assumption (the genetic variant increases the risk factor in all individuals in the population, or decreases the risk factor in all individuals), a local average causal effect can be estimated (Imbens and Angrist, 1994). However, local average causal effects may differ between valid IVs. We return to this point in the discussion.

### 2.2 Heterogeneity between causal estimates and clustered heterogeneity

**Fig. 2.**
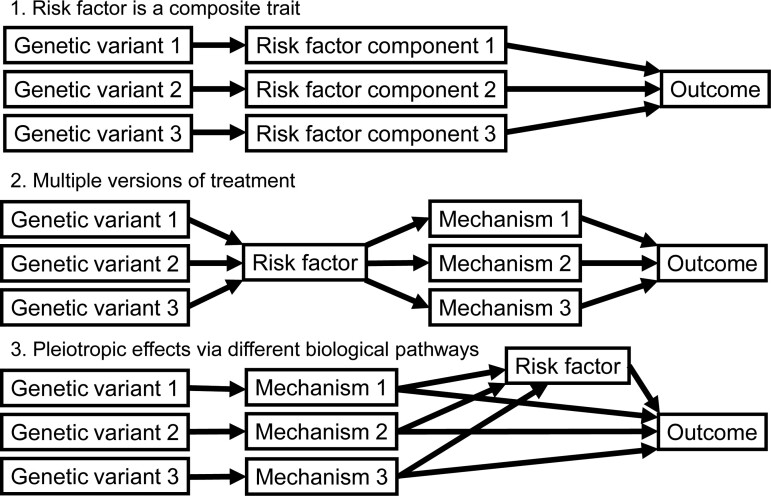
Scenarios that could lead to clustered heterogeneity, defined as the case where causal estimates from multiple variants tend towards a number of distinct values as the sample size increases. Clustered heterogeneity could arise in a number of ways: the mechanisms may represent distinct components of the risk factor, or distinct pathways by which the risk factor may influence the outcome, or intermediaries on the causal pathway from the genetic variant to the outcome

Even if the IV assumptions and parametric assumptions (1) and (2) are satisfied for each genetic variant, it is plausible that the variant-specific ratio estimates ${\hat{\theta }}_{j}$ differ by more than expected due to chance alone. We are particularly interested in the case where there are distinct values of the causal effect that are evidenced by multiple genetic variants, such that if the sample size were to tend towards infinity, the ratio estimates would tend towards a number of distinct values. We refer to this situation as clustered heterogeneity. Clustered heterogeneity is interesting to investigate as the identity of the genetic variants in the clusters may reveal information about causal pathways relating to the outcome. Figure 2 illustrates how different variants may be associated with the risk factor and outcome via different mechanisms. This situation could arise in a number of ways:

1. Risk factor is a composite trait: the risk factor is not a single entity, but in fact contains multiple components with distinct causal effects. For example, although serum cholesterol concentration can be expressed as a single measurement, evidence suggests that cholesterol carried by low-density lipoprotein particles has a different causal relationship to CAD risk compared with cholesterol carried by high-density lipoprotein (LDL) particles (Voight *et al.*, 2012).
2. Multiple versions of treatment: The risk factor can be intervened on in different ways, and each intervention leads to a different size of change in the outcome. For example, interventions to lower BMI via decreasing an individual’s caloric intake are likely to lead to less cardiovascular benefit compared with interventions to increase metabolic rate.
3. Pleiotropic effects via different biological pathways: Even if the risk factor is a single trait and there is a single version of treatment, genetic variants may associate with the risk factor via pleiotropic pathways, which may influence the outcome directly (i.e. not via the risk factor).

In the first two situations, identifying features of genetic variants in different clusters could help explain how the outcome is influenced by different components of the risk factor or different causal pathways from the risk factor, and hence inform our biological understanding of the causal relationship between the risk factor and outcome. In the third situation, the IV assumptions are violated, as the effects of the genetic variants on the outcome are not completely mediated via the risk factor, but are mediated via the pleiotropic variable. In this case, investigating traits that associate preferentially with variants in different clusters could identify intermediaries on the relevant causal pathway for each cluster.

In Supplementary Appendix Section SA, we provide some theoretical motivation that clustered heterogeneity arises if and only if genetic variants in the same cluster affect the outcome via the same distinct causal pathway, under assumptions of linearity and homogeneity. However, it is impossible to distinguish between the scenarios listed above on the basis of the genetic associations with the risk factor and outcome alone. In particular, it is not possible to distinguish situations 1 and 2 (in which the risk factor causally affects the outcome) from situation 3 (in which the effect on the outcome is via a pleiotropic mechanism). In the applied example, we perform a *post hoc* exploratory analysis to investigate whether there are traits that associate preferentially with variants in a given cluster, as an attempt to interpret the mechanism represented by the cluster. Such a trait may represent a pleiotropic variable that is influenced by variants in the cluster, or variable downstream of a specific causal mechanism by which the risk factor influences the outcome.

## 3 Algorithm

We proceed to introduce a statistical method for clustering causal estimates from different genetic variants. We suppose that there are *K *+* *2 disjoint clusters of genetic variants: *K* substantive clusters, a null cluster and a junk cluster. The substantive clusters $S_{1},\ldots ,S_{K}$ have means *θ_k_*, k=1,…,K. The null cluster *S*_0_ has mean $\theta_{0}=0$. The presence of the null cluster ensures that genetic variants which do not suggest a causal effect of the risk factor do not contribute to the estimates of the substantive cluster means. The junk cluster $S_{K+1}$ comprises all remaining genetic variables that are not members of the other clusters. The presence of the junk cluster ensures that genetic variants which do not fit into any of the substantive clusters do not contribute to the estimates of the substantive cluster means. Together, the null and junk clusters require there to be substantial evidence of similarity of estimates from several genetic variants to define a substantive cluster. This should minimize false-positive findings from the method. If we only used a single null cluster with a large variance and no junk cluster, then variants having estimates close to the null may be selected into one of the substantive clusters. However, there is no reason why different genetic variants having null associations with the outcome would share a common mechanism.

### 3.1 Mixture model

For each genetic variant j=1,…,J, we introduce a cluster allocation label *z_j_*, such that $z_{j}=k\Leftrightarrow G_{j}\in S_{k}$. For variants in the substantive and null clusters, we assume that the ratio estimate ${\hat{\theta }}_{j}$ for variant *j* in cluster *k* follows a normal distribution with mean *θ_k_* and standard deviation ${\hat{\sigma }}_{j}$, taken as the standard error of the *j*th ratio estimate:

(7) $${\hat{\theta }}_{j}|\{\Theta ,{\hat{\sigma }}_{j}^{2},z_{j}=k\}∼N\left(\theta_{k},{\hat{\sigma }}_{j}^{2}\right)\;\;\text{for}\;k=0,1,\ldots ,K,$$

where Θ is a vector of the cluster means. For simplicity, we do not account for uncertainty in the estimate of the standard error.

Following Crook *et al.* (2018), we assume ratio estimates for variants in the junk cluster follow a generalized t-distribution with degrees of freedom *ν* = 4, mean *μ* taken as the sample mean of all the ratio estimates ($\mu =\sum_{j=1}^{J}{\hat{\theta }}_{j}/J$), and scale parameter *ψ* taken as

(8) $$|{\hat{\theta }}_{\max}-{\hat{\theta }}_{\min}|+2{\hat{\sigma }}_{\max},$$

where ${\hat{\theta }}_{\max}$ is the maximum of the ratio estimates, ${\hat{\theta }}_{\min}$ is the minimum of the ratio estimates and ${\hat{\sigma }}_{\max}$ is the maximum of the standard errors of the ratio estimates. We discuss the specification of this distribution in Supplementary Appendix Section SB.

We obtain a mixture model for the ratio estimates ${\hat{\theta }}_{j}$:

(9) $$\begin{array}{c} p({\hat{\theta }}_{j}|\Theta ,{\hat{\sigma }}_{j}^{2})=\sum_{k=0}^{K+1}p({\hat{\theta }}_{j},z_{j}=k|\Theta ,{\hat{\sigma }}_{i}^{2})\\ =\sum_{k=0}^{K+1}p(z_{j}=k)p({\hat{\theta }}_{j}|\Theta ,{\hat{\sigma }}_{j}^{2},z_{j}=k)\\ =\pi_{0}\;\phi ({\hat{\theta }}_{j}|0,{\hat{\sigma }}_{j}^{2})+\sum_{k=1}^{K}\pi_{k}\;\phi ({\hat{\theta }}_{j}|\theta_{k},{\hat{\sigma }}_{j}^{2})\\ +\;\pi_{K+1}\;T({\hat{\theta }}_{j}), \end{array}$$

where *π_k_* is the mixture proportion for cluster *k*, $\phi (x|\mu ,\sigma^{2})$ denotes the univariate normal density evaluated at *x* with mean *μ* and variance $\sigma^{2}$, and T(x) denotes the generalized t-distribution evaluated at *x* with degrees of freedom *ν* = 4, and mean *μ* and scale parameter *ψ* as discussed above.

### 3.2 Parameter estimation via expectation maximization

The 2K+1 parameters *θ_k_* and *π_k_* (*K* cluster means and *K *+* *2 proportions, less one as the proportions must sum to one: $\sum_{k=0}^{K+1}\pi_{k}=1$) in Equation (9) are estimated via an expectation-maximization (EM) algorithm for a given number of substantive clusters *K*. We then estimate the number of substantive clusters.

The log-likelihood of the sample data $\hat{\theta }$ (the ratio estimates) is

(10) $$\begin{array}{c} \;\text{log}\;p_{\hat{\theta }\;|\;\{\theta ,\pi ,K\}}=\text{log}\;p\left(\hat{\theta }|\theta ,\pi ,\hat{\sigma },K\right)\\ =\sum_{j=1}^{J}\;\text{log}\;[\pi_{0}\;\phi \left({\hat{\theta }}_{j}|0,{\hat{\sigma }}_{j}^{2}\right)\\ +\sum_{k=1}^{K}\pi_{k}\;\phi \left({\hat{\theta }}_{j}|\theta_{k},{\hat{\sigma }}_{j}^{2}\right)+\pi_{K+1}\;T\left({\hat{\theta }}_{j}\right)]. \end{array}$$

We denote the maximum likelihood estimate (MLE) of the unknown parameters for a given *K* as $\left\{\theta_{K}^{*},\pi_{K}^{*}\right\}$. For ease of presentation, we drop the index *K* in this section.

We index each iteration of the EM algorithm by the variable *i* so that the pair $\left\{\theta^{\left(i\right)},\pi^{\left(i\right)}\right\}$ denotes estimates of the cluster means and mixture proportions at the *i*th iteration of the algorithm. We stop updating the parameters when the difference in log-likelihood between two iterations falls below a user-defined tolerance *δ*.

We describe the algorithm in three main steps: (i) an initialization step to obtain initial values of the parameters, and (ii) an expectation step and (iii) a maximization step to update the parameter values.

#### 3.2.1 Initialization step

Reliable estimation of the MLE might depend crucially on the initialization of the parameters. To mitigate sensitivity to the initialization, our algorithm computes multiple estimates of the MLE over various initializations of the parameters. When *K *>* *0, for each initialization, we generate values for the cluster means $\left\{\theta^{\left(0\right)}\right\}$ via a *k*-means clustering of the data $\left\{\hat{\theta }\right\}$. We note this method does not account for the uncertainty in the ratio estimates. The initial mixture proportions $\left\{\pi^{\left(0\right)}\right\}$ are computed by first randomly drawing values for the proportion of samples in the null and junk mixtures $\left\{\pi_{0}^{\left(0\right)},\pi_{K+1}^{\left(0\right)}\right\}$ from the range (0.05,0.4). This ensures that the prior probability of belonging to either the null or junk cluster is at least 10% and at most 80%. The remaining parameters $\left\{\pi_{1}^{\left(0\right)},\ldots ,\pi_{K}^{\left(0\right)}\right\}$ are then computed as the proportion of observations assigned to each of the *K* clusters from the *k*-means analysis multiplied by $\left(1-\pi_{0}^{\left(0\right)}-\pi_{K+1}^{\left(0\right)}\right)$.

#### 3.2.2 Expectation step

Let ***Z*** denote the collection of cluster allocation labels $\left\{z_{1},z_{2},\ldots ,z_{J}\right\}$ for the variants j=1,2,…,J. Before updating the unknown parameters in the maximization step, we first evaluate:

(11) $$\begin{array}{c} E_{Z|\hat{\theta },\theta^{\left(i\right)},\pi^{\left(i\right)}}\left[\text{log}\;p_{\hat{\theta },Z\;|\;\{\theta ,\pi \}}\right]\\ =\sum_{j=1}^{J}\sum_{z_{j}=0}^{K+1}r_{ijz_{j}}\;\text{log}[I\left(z_{j}\leq K\right)\pi_{z_{j}}\phi \left({\hat{\theta }}_{j}|\theta_{z_{j}},{\hat{\sigma }}_{j}^{2}\right)\\ +I\left(z_{j}=K+1\right)\pi_{K+1}T\left({\hat{\theta }}_{j}\right)] \end{array}$$

which requires computation of the conditional allocation probabilities for each observation:

(12) $$\begin{array}{c} r_{\mathrm{ijk}}=p\left(z_{j}=k|\hat{\theta },\theta^{\left(i\right)},\pi^{\left(i\right)}\right)\\ =\frac{\pi_{k}^{\left(i\right)}\phi ({\hat{\theta }}_{j};\theta_{k}^{\left(i\right)},{\hat{\sigma }}_{j}^{2})}{\pi_{0}^{\left(i\right)}\phi \left({\hat{\theta }}_{j};0,{\hat{\sigma }}_{j}^{2}\right)+\sum_{k=1}^{K}\pi_{k}^{\left(i\right)}\phi ({\hat{\theta }}_{j};\theta_{k}^{\left(i\right)},{\hat{\sigma }}_{j}^{2})+\pi_{K+1}^{\left(i\right)}T\left({\hat{\theta }}_{j}\right)} \end{array}$$

for k=1,…,K, and:

(13) $$r_{\mathrm{ijk}}=\frac{\pi_{K+1}^{\left(i\right)}T\left({\hat{\theta }}_{j}\right)}{\pi_{0}^{\left(i\right)}\phi \left({\hat{\theta }}_{j};0,{\hat{\sigma }}_{j}^{2}\right)+\sum_{k=1}^{K}\pi_{k}^{\left(i\right)}\phi ({\hat{\theta }}_{j};\theta_{k}^{\left(i\right)},{\hat{\sigma }}_{j}^{2})+\pi_{K+1}^{\left(i\right)}T\left({\hat{\theta }}_{j}\right)}$$

for k=K+1. The *r_ijk_* are sometimes referred to as the responsibilities of the *k*th component for the *j*th observation (evaluated here at the *i*th iteration of the EM algorithm).

#### 3.2.3 Maximization step

Updates for the unknown parameters are obtained by maximizing Equation (11). For the cluster means *θ_k_*, we solve the system of equations

$$\sum_{j=1}^{J}r_{\mathrm{ijk}}\frac{\partial }{\partial \theta_{k}}\text{log}\;\phi \left({\hat{\theta }}_{j}|\theta_{k},{\hat{\sigma }}_{j},K\right)=0,\;k=1,2,\ldots ,K.$$

Re-arranging for *θ_k_*, and taking this as the update $\theta_{k}^{\left(i+1\right)}$, returns

(14) $$\theta_{k}^{\left(i+1\right)}=\frac{\sum_{j=1}^{J}r_{\mathrm{ijk}}{\hat{\theta }}_{j}{\hat{\sigma }}_{j}^{-2}}{\sum_{j=1}^{J}r_{\mathrm{ijk}}{\hat{\sigma }}_{j}^{-2}},\;k=1,2,\ldots ,K.$$

The updated Equation (14) resembles the inverse-variance weighted (IVW) estimate of the causal effect of the risk factor on the outcome (Burgess *et al.*, 2013; Johnson, 2013). For a cluster *S* of ratio estimates that target the same causal parameter (i.e. the ratio estimates tend to the same causal parameter as the sample size increases), the IVW estimate is the best linear unbiased estimate (BLUE) of this parameter (Wooldridge, 2009). It is given by

$${\hat{\theta }}_{\mathrm{IVW}}(S)=\frac{\sum_{j\in S}{\hat{\theta }}_{j}{\hat{\sigma }}_{j}^{-2}}{\sum_{j\in S}{\hat{\sigma }}_{j}^{-2}}.$$

Comparing the above with Equation (14), it follows that the EM update $\theta_{k}^{\left(i+1\right)}$ is a re-weighted IVW estimate for the parameter *θ_k_*. The weights are multiplied by the responsibilities *r_ijk_* which penalize the influence of observations that are centred away from the current estimate of $\theta_{k}^{\left(i\right)}$ and/or are highly diffuse (i.e. ${\hat{\sigma }}_{j}$ is large). In the large sample limit, as ${\hat{\sigma }}_{j}\to 0$ for each *j*, it follows from Equation (12) that

(15) $$r_{*jk}=\{\begin{array}{c} 1,\;\text{if}\;{\hat{\theta }}_{j}\to \theta_{k}\\ 0,\;\text{if}\;{\hat{\theta }}_{j}↛↛\theta_{k} \end{array},$$

 

(16) $$\Rightarrow \;\;\theta_{k}^{*}={\hat{\theta }}_{\mathrm{IVW}}(S_{k})\;\;\;\;k=1,2,\ldots ,K,$$

where $r_{*jk}$ denotes the responsibility of the *k*th component for the *j*th observation computed at an iteration of the EM algorithm in which the MLE is achieved.

The updated equations for the mixture proportions *π_k_* are obtained by first modifying Equation (11) to account for the constraint that $\sum_{k=0}^{K+1}\pi_{k}=1$ by introducing a Lagrange multiplier, and then maximizing. This is equivalent to solving the following system of equations:

(17) $$\begin{array}{c} \frac{\partial }{\partial \pi_{k}}\left(\sum_{j=1}^{J}r_{\mathrm{ijk}}\;\text{log}\;\pi_{k}+\lambda \left(\sum_{k=0}^{K+1}\pi_{k}-1\right)\right)=0,\\ \;\Rightarrow \;\lambda =J\;\text{and}\;\pi_{k}^{\left(i+1\right)}=\frac{\sum_{j=1}^{J}r_{\mathrm{ijk}}}{J}, \end{array}$$

for k=0,1,…,K+1, where *λ* denotes the Lagrange multiplier.

### 3.3 Determining the number of clusters

We first calculate the MLEs $\left\{\theta_{K}^{*},\pi_{K}^{*}\right\}$ for each value of K∈{0,1,…,J} possible substantive clusters present in the data. We estimate the number of substantive clusters $K^{*}$ by minimizing the Bayesian information criterion (BIC):

(18) $$\begin{array}{c} \underset{K\in \{0,1,\ldots ,J\}}{\min}BIC\left(K\right)\\ =\underset{K\in \{0,1,\ldots ,J\}}{\min}\left((2K+1)\;\text{log}\;J-2\;\text{log}\;p_{\hat{\theta }\;|\;\{\theta_{K}^{*},\pi_{K}^{*}\}}\right)\\ =\mathrm{BIC}(K^{*}). \end{array}$$

This helps to avoid overparameterization, as the BIC penalizes models which assume that the data are generated from larger numbers of underlying clusters.

Pseudocode outlining all steps in the MR-Clust algorithm is given in Algorithm 1. In practice, if *J* is large, then we calculate the MLE and BIC for increasing values of *K* starting at zero, and stop the algorithm once there is evidence that the BIC is increasing monotonically with *K*.

**Algorithm 1 MR-Clust – Expectation Maximization (EM) Algorithm**

Require: global convergence parameter *δ*, number of initializations *I*, number of variants *J*.

1: **for**  K=0,1,…,J  **do**

2:  **for** initialization ι=1,2,…,I  **do**

3:   generate: cluster means $\theta_{K}^{\left(0\right)}$ and mixture proportions $\pi_{K}^{\left(0\right)}$.

4:     compute: $r_{0jk},\;j=1,2,\ldots ,J;k=0,1,\ldots ,K+1.$

5:     update: $\left\{\theta_{k}^{\left(1\right)},\pi_{k}^{\left(1\right)}\right\}\;$  k=0,1,…,K+1.

6:     compute: $\text{log}\;\left(p_{\hat{\theta }\;|\;\left\{\theta_{K}^{\left(1\right)},\pi_{K}^{\left(1\right)}\right\}}/p_{\hat{\theta }\;|\;\left\{\theta_{K}^{\left(0\right)},\pi_{K}^{\left(0\right)}\right\}}\right)$

7:     set: *i *=* *1.

8:   **while**  $\text{log}\;\left(p_{\hat{\theta }\;|\;\left\{\theta_{K}^{\left(i\right)},\pi_{K}^{\left(i\right)}\right\}}/p_{\hat{\theta }\;|\;\left\{\theta_{K}^{\left(i-1\right)},\pi_{K}^{\left(i-1\right)}\right\}}\right)>\delta$  **do**

9:     compute: *r_ijk_*  j=1,2,…,J;k=0,1,…,K+1.

10:   update: $\left\{\theta_{k}^{\left(i+1\right)},\pi_{k}^{\left(i+1\right)}\right\}$     k=0,1,…,K+1.

11:   set: i→i+1.

12:   compute: $\text{log}\;\left(p_{\hat{\theta }\;|\;\left\{\theta_{K}^{\left(i\right)},\pi_{K}^{\left(i\right)}\right\}}/p_{\hat{\theta }\;|\;\left\{\theta_{K}^{\left(i-1\right)},\pi_{K}^{\left(i-1\right)}\right\}}\right)$.

13:  **end while**

14:   store: $\{\theta_{K;\iota }^{\left(*\right)},\pi_{K;\iota }^{\left(*\right)}\}=\{\theta_{K}^{\left(i\right)},\pi_{K}^{\left(i\right)}\}.$

15:  **end for**

16:  compute: $\left\{\theta_{K;\iota^{*}}^{\left(*\right)},\pi_{K;\iota^{*}}^{\left(*\right)}\right\}$

 where $\max_{\iota }\;\text{log}\;p_{\hat{\theta }\;|\;\left\{\theta_{K;\iota }^{\left(*\right)},\pi_{K;\iota }^{\left(*\right)}\right\}}=\text{log}\;p_{\hat{\theta }\;|\;\left\{\theta_{K;\iota^{*}}^{\left(*\right)},\pi_{K;\iota^{*}}^{\left(*\right)}\right\}}$.

17:  store: $\left\{\theta_{K}^{\left(*\right)},\pi_{K}^{\left(*\right)}\}=\{\theta_{K;\iota^{*}}^{\left(*\right)},\pi_{K;\iota^{*}}^{\left(*\right)}\right\}$.

18: **end for**

**Output:**  $\left\{K^{*},\theta_{K^{*}}^{\left(*\right)},\pi_{K^{*}}^{\left(*\right)}\right\}$

  where $\mathrm{BIC}(K^{*})=\min_{K=0,1,\ldots ,J}BIC(K)$.

## 4 Implementation

We perform a simulation study, comparing results from our MR-Clust method to those obtained using three comparison methods. The Mclust method (Scrucca *et al.*, 2016) is a popular model-based clustering, classification and density estimation method based on finite normal mixture modelling. Unlike MR-Clust, Mclust does not account for observation-specific uncertainty in the ratio estimates when assigning observations to clusters, but rather estimates a cluster-specific variance parameter for each cluster. It also does not incorporate null or junk clusters. We also compare results against the T-Augmented Gaussian Mixture model (TAGM) method, an extension of Mclust to include a junk component (Crook *et al.*, 2018). The original version of TAGM was a semi-supervised method, which is relevant to its initial application to proteomic data, but is not relevant here. We have adapted TAGM to exclude this aspect of the method for comparison. We also compare with a version of the MR-Clust method without a junk cluster. Unless indicated otherwise, all references to the MR-Clust method relate to the implementation of the method with a junk cluster.

The four methods are summarized in Table 1. By comparing these methods, we show how features of the MR-Clust method, the null and junk clusters and allowance for differential uncertainty in the observations, help MR-Clust to correctly identify the number of clusters present in the data. We then perform an applied analysis to demonstrate the method in practice. Unless indicated otherwise, all references to the MR-Clust method relate to the implementation of the method with a junk cluster.

**Table 1.** Summary of methods compared in the simulation study and applied example

Method	Allows for differential uncertainty?	Includes junk cluster?	Includes null cluster?
Mclust	No	No	No
TAGM	No	Yes	No
MR-Clust without junk	Yes	No	Yes
MR-Clust with junk	Yes	Yes	Yes

### 4.1 Simulation: set-up and scenarios

We simulate data on genetic associations with a risk factor (${\hat{\beta }}_{Xj}$) and with an outcome (${\hat{\beta }}_{Yj}$) for 90 genetic variants indexed by *j*. These associations imitate coefficient estimates from linear regression of a continuous variable with variance 1 on a single nucleotide polymorphism (SNP). A SNP can be thought of as a binomial random variable taking values 0, 1, 2, representing the number of minor alleles inherited from one’s parents at a particular location of the genetic code.

(19) $$\begin{array}{c} {\hat{\beta }}_{Xj}∼N\left(\mu_{\beta_{Xj}},\frac{1}{N\;{\text{MAF}}_{j}(1-{\text{MAF}}_{j})}\right),\\ {\hat{\beta }}_{Yj}∼N\left(\theta_{j}\;{\hat{\beta }}_{Xj},\frac{\tau }{N\;{\text{MAF}}_{j}(1-{\text{MAF}}_{j})}\right),\\ \mu_{\beta_{Xj}}∼N\left(0,1\right),\\ MAF_{j}∼\text{Uniform}\left(0.05,0.5\right),\\ \text{se}({\hat{\beta }}_{Xj})=\text{se}({\hat{\beta }}_{Yj})=\sqrt{\frac{1}{N\;{\text{MAF}}_{j}(1-{\text{MAF}}_{j})}}, \end{array}$$

where *N* is the notional sample size in which the genetic associations are estimated, $\mu_{\beta_{Xj}}$ is the true genetic effect on the risk factor for variant *j*, *θ_j_* is the causal effect for variant *j*, *MAF_j_* is the minor allele frequency of variant *j*, *τ* is an overdispersion parameter, and all distributions are sampled independently.

We consider 4 scenarios, and simulate 1000 datasets in each scenario. In Scenarios 1 and 2, there are no non-null clusters, and $\theta_{j}=0$ for all *j*. In Scenarios 3 and 4, there are three non-null clusters $\theta_{j}=0.4$ for $j=1,\ldots ,10,\;\theta_{j}=-0.4$ for $j=11,\ldots ,30,\;\theta_{j}=0.8$ for j=31,…,70, a junk cluster in which the *θ_j_* are drawn from a standard normal distribution for j=71,…,80, and a null cluster $\theta_{j}=0$ for j=81,…,90. In Scenarios 1 and 3, we set *τ* = 1 and in Scenarios 2 and 4, we set *τ* = 2. Scenario 1 represents a null scenario, in which all genetic variants should be included in the null cluster. Scenario 2 represents a variance-inflated null scenario, in which all genetic variants should be included in either the null or junk clusters. These scenarios are considered to assess whether the methods find spurious clusters where they do not truly exist. In Scenarios 3 and 4, the methods should find three clusters of 10, 20 and 40 variants each, and the other 20 variants should be included in either the null or junk cluster. We repeat the simulation for sample sizes of *N *=* *1000 and *N *=* *5000. Parameter values are displayed in Table 2.

**Table 2.** Number of variants and causal effect in each cluster for the simulation study

Scenarios	Number of variants					Cluster causal effect (*θ_j_*)				
	Null	Junk	Cluster 1	2	3	Null	Junk	Cluster 1	2	3
1 and 2	90	0	0	0	0	0	–	–	–	–
3 and 4	10	10	10	20	40	0	∼N(0,1)	0.4	–0.4	0.8

*Note*: In Scenarios 1 and 3, there is no excess heterogeneity in the genetic associations with the outcome (*τ* = 1); in Scenarios 2 and 4, there is excess heterogeneity (*τ* = 2).

### 4.2 Simulation results

**Fig. 3.**
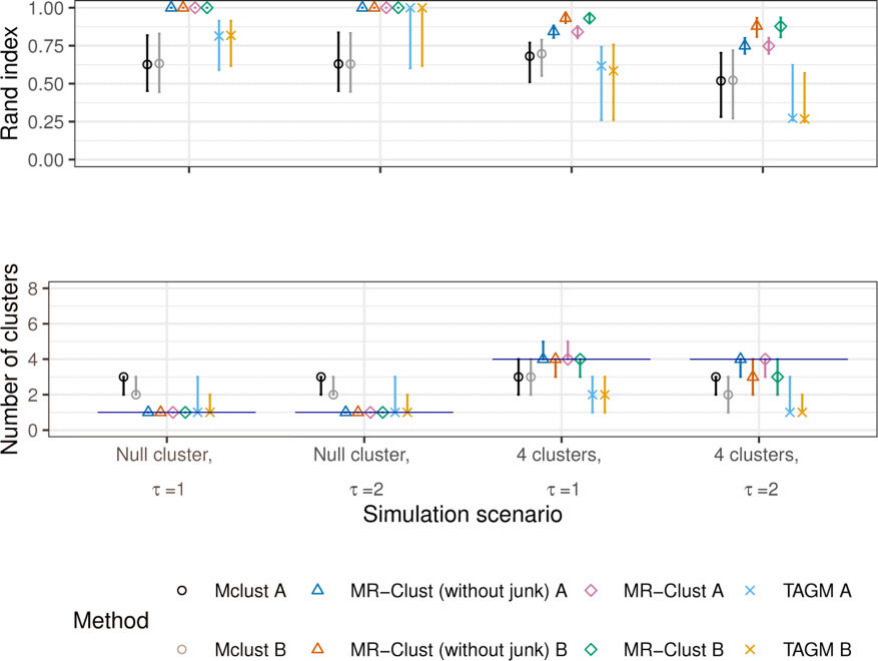
Results from the simulation study with sample size *N *=* *1000 for MR-Clust (with and without junk cluster), Mclust and TAGM methods under four scenarios for the Rand index (top panel) and the number of clusters identified (bottom panel). Points represent median values across simulated datasets, and vertical bars represent the first and ninth deciles. The horizontal line in the bottom panel represents the true number of clusters in each scenario. Two versions of each method are presented: (A) each variant is assigned to the cluster with the greatest conditional probability; (B) variants are only assigned to a cluster if the conditional probability is ≥0.8 and clusters are only displayed if at least 4 variants are assigned to the cluster

**Fig. 4.**
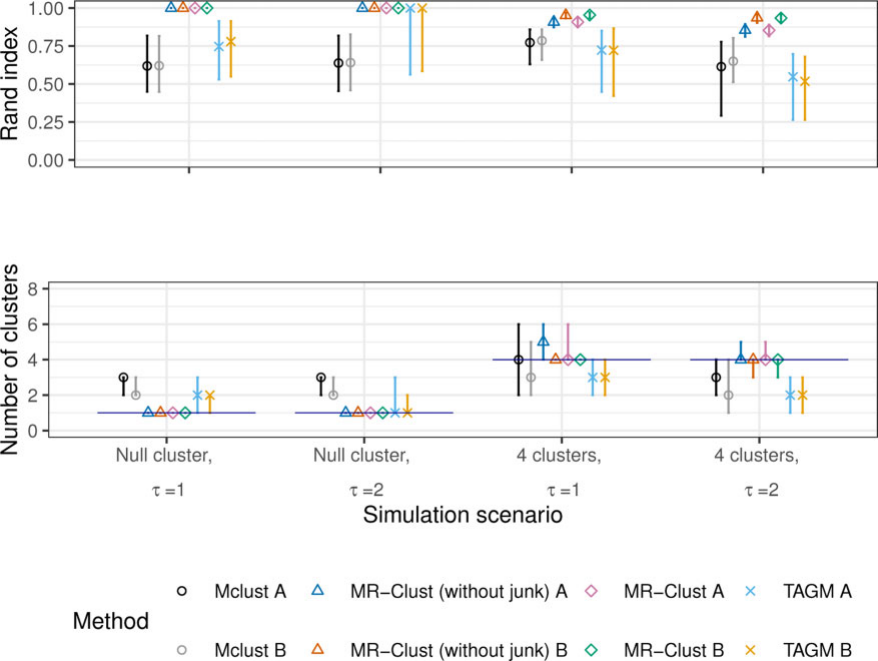
Results from the simulation study with sample size *N *=* *5000 for MR-Clust (with and without junk cluster), Mclust and TAGM methods under four scenarios for the Rand index (top panel) and the number of clusters identified (bottom panel). Points represent median values across simulated datasets, and vertical bars represent the first and ninth deciles. The horizontal line in the bottom panel represents the true number of clusters in each scenario. Two versions of each method are presented: (A) each variant is assigned to the cluster with the greatest conditional probability; (B) variants are only assigned to a cluster if the conditional probability is ≥0.8 and clusters are only displayed if at least 4 variants are assigned to the cluster

Results from the simulation study are displayed in Figure 3 for a sample size of *N *=* *1000 and Figure 4 for a sample size of *N *=* *5000. We present the Rand index (top panel), which measures the similarity between the true and estimated allocations into clusters (Rand, 1971), and the number of clusters identified by each method (bottom panel). For comparability, for the MR-Clust method, we show the number of substantive clusters plus one for the null cluster, as this is the number of clusters in the data as well as the number that the Mclust and TAGM methods should detect. We compare two versions of each method: (A) each variant is assigned to the cluster with the greatest conditional probability (responsibility); and (B) variants are only assigned to a cluster if the conditional probability is ≥0.8, otherwise they are unassigned, and only substantive clusters with at least 4 assigned variants are reported. Version (B) is recommended to discourage the reporting of clusters that are evidenced by only a few variants, which therefore may well be spurious. The thresholds of 0.8 for the probability and 4 for the number of variants are arbitrary choices, but gave good performance in the simulation setting. In version (A), all variants contribute to the calculation of the Rand index. In version (B), only variants in a cluster of at least 4 assigned variants contribute. In Scenarios 3 and 4, genetic variants in the junk cluster do not contribute to the calculation of the Rand index. This is to ensure a fair comparison between methods that include a junk cluster and those that do not.

In Scenarios 1 and 2, both versions of MR-Clust with and without the junk cluster perform well, identifying spurious clusters in less than 10% of simulated datasets. In contrast, both the Mclust and TAGM methods identify spurious clusters more often. The Rand index is close or equal to 1 for both versions of the MR-Clust method for all simulated datasets, but substantially lower for the Mclust and TAGM methods. In Scenarios 3 and 4, MR-Clust continued to perform well in both scenarios, regularly identifying all four clusters in the data. Version (A) of the MR-Clust method without the junk cluster occasionally detected an extra cluster, and version (B) with and without a junk cluster sometimes failed to detect a cluster in simulations with *N *=* *1000. However, the Rand index was consistently high for both MR-Clust methods. In contrast, the Mclust and TAGM methods had much lower Rand indices, and regularly failed to identify all four clusters.

There was little difference in performance between the MR-Clust method with a junk cluster and without a junk cluster. This is for two main reasons. First, junk variants do not contribute to the Rand index. Hence the method with a junk cluster is not commended for correctly assigning these variants to the junk cluster rather than to a substantive cluster. Second, junk variants are unlikely to have similar estimates. Hence, it is unlikely that the presence of junk variants will cause the method to incorrectly estimate the number of clusters. The presence of the junk cluster reduces the number of false-positive members of a cluster by providing a fixed barrier to cluster entry. If evidence that a variant belongs to a cluster does not reach the necessary threshold, then rather than assigning it to the nearest cluster, it is allowed to not belong to any substantive cluster.

**Fig. 5.**
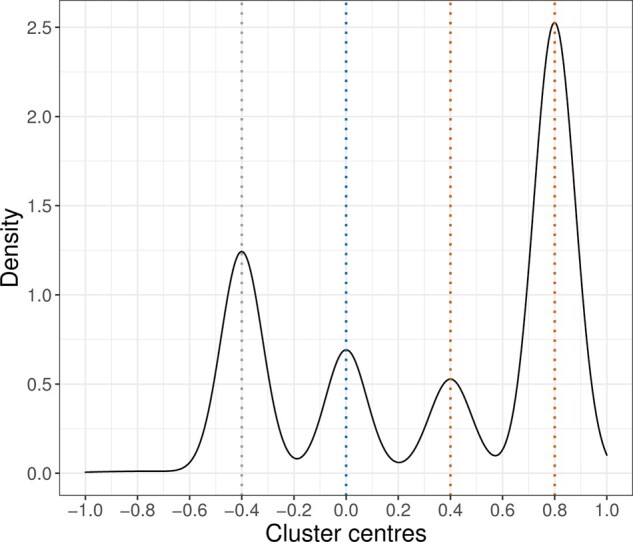
Kernel-weighted density plot of cluster means identified by MR-Clust method in simulation scenario 4. Dashed vertical lines represent the true values of the cluster means

To further illustrate the MR-Clust method, we plot a kernel-weighted density estimate of the distribution of estimated cluster means across the 1000 datasets in Scenario 4 with a sample size of *N *=* *5000 (Fig. 5). On average, MR-Clust identified the correct cluster means at {−0.4,0,0.4,0.8} as well the correct proportions of variants belonging to each cluster. We also plot the value of the log-likelihood at successive iterations of the EM algorithm corresponding to 6 initializations of the parameters for a selected dataset generated under scenario 4 (Supplementary Fig. SA1). In this example, the EM algorithm converged to different values of the log-likelihood between the initializations. This indicates some sensitivity of the method to the initial choice of mixture proportions and cluster means, and motivates our use of multiple initializations in the algorithm. We investigated this property across a range of further datasets and simulation scenarios, and usually found negligible differences in MLEs across initializations. However, it is worth checking convergence carefully in practice.

### 4.3 Applied example: blood pressure and coronary artery disease risk

We illustrate our method by considering the relationship between blood pressure and coronary artery disease (CAD) risk. Blood pressure is a heritable trait that is influenced by multiple biological pathways (Evangelou *et al.*, 2018; The International Consortium for Blood Pressure Genome-Wide Association Studies, 2011). Elevated blood pressure is considered to be a major risk factor for cardiovascular disease. We assess evidence of clustered heterogeneity in Mendelian randomization analyses for the causal effects of three blood pressure traits on CAD risk: systolic blood pressure (SBP), diastolic blood pressure (DBP) and pulse pressure (PP).

Genetic associations with the blood pressure traits were obtained from the International Consortium for Blood Pressure, and were estimated in 299 024 participants of European ancestry (Evangelou *et al.*, 2018). To avoid genetic associations being inflated due to winner’s curse, we only considered genetic variants that had been demonstrated to be associated with any blood pressure trait in a previous discovery genome-wide association study (GWAS) at a genome-wide level of significance ($P<5\times 10^{-8}$). For the analysis of each of the three blood pressure traits, we included all variants additionally associated with the trait under analysis (i.e. SBP, DBP or PP) in a replication dataset at a *P*-value threshold of $10^{-5}$. Variants were pruned to independence based on a distance threshold; only one variant was included in the analysis per gene region. The variants were all independently distributed ($r^{2}<0.01$). For SBP, 121 variants were included in the analysis; for DBP, 119 variants and for PP, 85 variants. Genetic associations with CAD risk were obtained from a meta-analysis of 122 733 cases and 424 528 controls primarily of European descent from the CARDIoGRAMplusC4D consortium and UK Biobank (van der Harst and Verweij, 2018). As the variants are strongly associated with the exposure, any bias due to the small degree of sample overlap between the risk factor and outcome datasets will be minimal (Burgess *et al.*, 2016a). We applied the MR-Clust method to assess evidence of clustered heterogeneity for each of the three blood pressure traits on CAD risk separately.

#### 4.3.1 Results

**Fig. 6.**
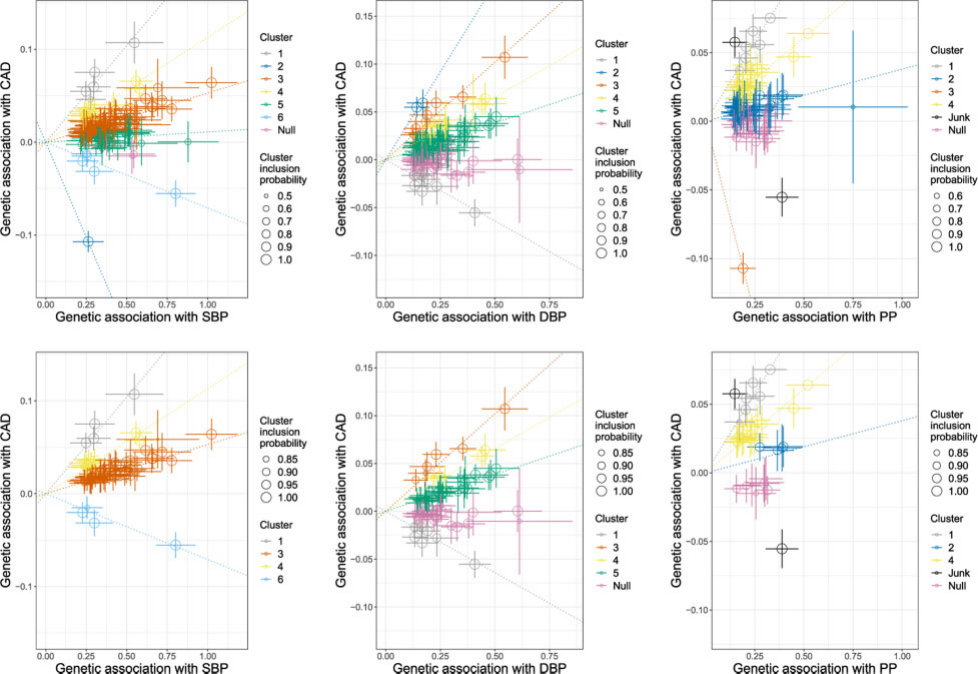
Genetic associations with blood pressure traits (mmHg) and coronary artery disease risk (log odds) per additional blood pressure-increasing allele. Each genetic variant is represented by a point. Error bars are 95% confidence intervals for the genetic associations. Colours represent the clusters, and dotted lines represent the cluster means. Top row: method version (A)—each variant is assigned to the cluster with the greatest conditional probability. Bottom row: method version (B)—variants are only assigned to a cluster if the conditional probability is ≥0.8, and clusters are only displayed if at least 4 variants are assigned to the cluster. Left column: systolic blood pressure; middle column: diastolic blood pressure; and right column: pulse pressure

Results are displayed in Figure 6. An extract of the results is shown in Table 3, and full results in Supplementary Table SA2. Following the simulation study, we present results according to version (A) of the method (all variants assigned to a cluster: top panels) and version (B) (variants assigned to a cluster if conditional probability ≥0.8, only clusters with at least 4 variants reported: bottom panels). Although the number of clusters identified varies between SBP and DBP for version (A) of the method, four clusters are reported in version (B) of the method for both traits. This is despite the number and identity of variants varying between the analyses. The largest cluster suggests a positive causal effect of blood pressure on CAD risk. There are also two clusters suggesting a stronger positive causal effect and one suggesting a weak negative effect. For PP, all three substantive clusters in version (B) suggest a positive effect on CAD risk. This suggests the presence of multiple mechanisms by which blood pressure influences CAD risk.

**Table 3.** Extract of summary of genetic variants and assignment to clusters

rsid	SBP				DBP				PP			
	Cluster	Mean	Estimate	SE	Cluster	Mean	Estimate	SE	Cluster	Mean	Estimate	SE
rs3184504	4	0.113	0.100	0.010	4	0.131	0.131	0.012	–	–	0.390	0.037
rs12579720	4	0.113	0.119	0.021	4	0.131	0.132	0.024	–	–	–	–
rs12940887	4	0.113	0.140	0.020	4	0.131	0.150	0.022	–	–	–	–
rs6797587	4	0.113	0.124	0.019	4	0.131	0.157	0.024	–	–	–	–
rs2521501	4	0.113	0.118	0.011	3	0.204	0.186	0.018	4	0.231	0.273	0.026
rs1063281	4	0.113	0.137	0.025	3	0.204	0.237	0.043	–	–	–	–
rs112557609	4	0.113	0.121	0.025	–	–	–	–	–	–	0.180	0.037
rs2972146	4	0.113	0.113	0.022	–	–	0.202	0.039	–	–	–	–

*Note*: Separate analyses for systolic blood pressure (SBP), diastolic blood pressure (DBP) and pulse pressure (PP): cluster number (greatest conditional probability), cluster mean, ratio estimate for that variant and its standard error. The full results for all 180 variants are in Supplementary Table SA2. Dashes either indicate that the variant was not associated with the relevant blood pressure trait at $P<10^{-5}$ (if the estimate is absent), or that the variant was assigned to the null or junk cluster (if the estimate is present).

We also performed analyses for SBP using the Mclust and TAGM methods (Supplementary Fig. SA2). Each of the methods identified the largest cluster that was also found by the MR-Clust method. However, both methods combined all other variants into a single diffuse cluster, despite these variants clearly not belonging to a single cluster. This illustrates the value of using the variant-specific standard errors in judging whether variants are compatible with cluster membership, rather than estimating a cluster-specific heterogeneity without reference to these standard errors.

#### 4.3.2 Hypothesis-generating search for causal mechanism

To demonstrate how clustering can reveal biological mechanisms in the data, we focused on the genetic variants in the clusters for SBP and DBP with a negative effect on CAD risk, and performed a *post hoc* hypothesis-generating search of traits that associate with variants in these cluster as an exploratory analysis. We consider this cluster as it is smaller than the two positive clusters, and therefore more plausible that a single mechanism may be driving cluster membership for the majority of variants. In total, 10 genetic variants were assigned to this cluster with a conditional probability ≥0.8 in either the SBP or DBP analysis (Supplementary Table SA3). We looked up genetic associations in PhenoScanner, a database of genetic associations with traits and diseases (Staley *et al.*, 2016). For each trait in turn, we considered whether each variant was associated with that trait at $P<10^{-5}$ and $P<10^{-8}$, and report the true-positive rate (the proportion of variants in the cluster associated with the trait) and false-positive rate (the proportion of variants not in the cluster associated with the trait). This functionality is built into the *mrclust* software package. In total, we considered 3269 traits, although this includes several repeated or synonymous traits, and blood pressure traits. Also, some traits only had association estimates for a limited number of variants. This makes it difficult to correct for multiple comparisons. We therefore present results under the caveat that no correction has been attempted.

Results are shown in Table 4. The trait ‘Trunk fat percentage’ was associated with 5 out of the 9 variants in the cluster that were present in the dataset (true positive = 0.555), and only 14 out of the 169 variants not in the cluster (false positive = 0.083). Similarly ‘Impedance of arm right’ was associated with 4 out of 9 variants in the cluster (true positive = 0.444), and 15 out of the 169 variants not in the cluster (false positive = 0.089). Impedance is a measure of electrical resistance. It is greater when the body part has a higher fat percentage. At a threshold of $P<10^{-8}$, ‘Arm fat percentage’ was associated with 3 out of 9 variants in the cluster (true positive = 0.333) and only 4 out of the 169 variants not in the cluster (false positive = 0.024).

**Table 4.** Traits from hypothesis-generating search of predictors of cluster membership for cluster with negative causal effect

Trait	Threshold	True positives (%)	False positives (%)	*P*-value
Trunk fat percentage	$10^{-5}$	5/9 (55.5%)	14/169 (8.3%)	0.0022
Impedance of arm	$10^{-5}$	4/9 (44.4%)	15/169 (8.9%)	0.0239
Arm fat percentage	$10^{-8}$	3/9 (33.3%)	4/169 (2.4%)	0.0001

*Note*: True positives is the fraction of variants in the cluster that are associated with the trait at a *P*-value below the threshold. False positives is the fraction of variants not in the cluster (i.e. in any other cluster or not in a cluster at all) that are associated with the trait at a *P*-value below the threshold. The *P*-values in the rightmost column are for the hypothesis that association with the trait is independent of cluster membership. *P*-values are calculated by exact computation from the relevant hypergeometric distribution. We note that while in total 10 variants were assigned to the target cluster, 1 variant was not present in UK Biobank, the dataset in which the associations with these particular traits were all estimated. Hence the number of true positives is a fraction of 9, the number of variants present in UK Biobank.

This suggests that while most biological mechanisms associated with increased blood pressure lead to increased CAD risk, there also may be a biological mechanism associated with decreased blood pressure that leads to increased CAD risk. This mechanism relates to measures of adiposity and fat distribution. However, the directions of association with the adiposity measures were not consistent across variants in the cluster (Supplementary Table SA3).

We performed a multivariable Mendelian randomization analysis for SBP on CAD risk additionally adjusting for body mass index to assess mediation of the causal effect of SBP via adiposity (Burgess *et al.*, 2017). The coefficient for body mass index was imprecisely estimated and not significantly different from zero, suggesting that body mass index is not a strong mediator of the effect of SBP on CAD risk. Additionally, as a negative control, we searched whether variants in the null cluster were associated preferentially with any trait. No traits were preferentially associated with variants in the null cluster, confirming our view that there is no reason why variants in the null cluster should share a common mechanism.

We also performed the contamination mixture method for SBP on CAD risk. This is a method for Mendelian randomization that allows the possibility of multiple causal estimates in the analysis of a single risk factor and outcome, but does not attempt to cluster genetic variants (Burgess *et al.*, 2020). The main utility of the contamination mixture method is to provide a robust estimate of the causal effect evidenced by the largest subset of genetic variants. The log-likelihood function from this method is plotted in Supplementary Figure SA3. In this case, the log-likelihood is unimodal. While there is possibly a secondary peak in the log-likelihood at a negative value of the causal estimate, this is not clear. This result provides empirical evidence of the superiority of MR-Clust over the contamination mixture method for detecting multiple causal estimates.

### 4.4 Applied example: HDL-cholesterol and coronary artery disease risk

As a further example, we applied the MR-Clust method to an example of HDL cholesterol and CAD risk that we have considered previously (Burgess *et al.*, 2020). Results from the MR-Clust method are displayed in Supplementary Figure SA4. The method identified three clusters of variants: two with negative causal estimates, and one with a positive causal estimate. This is similar to results from the contamination mixture method, which also had two negative maxima, although it was not able to identify the cluster with a positive causal estimate.

## 5 Discussion

In this article, we have discussed how causal estimates based on different genetic variants in a Mendelian randomization investigation could differ. In particular, we have introduced the notion of clustered heterogeneity, and described how variants that influence the risk factor in different ways could target distinct causal effect parameters. We have introduced the MR-Clust method that detects clusters of variants having similar causal estimates. There are several distinguishing features of this method: it accounts for differential uncertainty in the causal estimates, and it includes null and junk clusters, so that variants are only included in a substantive cluster if there is strong evidence that they belong to that cluster. We demonstrated the benefits of these features in a simulation study, showing how our method outperforms clustering methods that do not have these features. Finally, we illustrated an application of the method to analyse the causal effect of blood pressure on CAD risk, demonstrating the existence of clusters of genetic variants in an empirical example.

In developing this method, our strong concern was to avoid finding spurious clusters of genetic variants that arise due to the chance similarity of causal estimates from different variants. For this reason, we recommend a conservative implementation of the method (version B), only assigning a variant to a cluster if the conditional probability of cluster assignment is ≥0.8, and only reporting a cluster if at least 4 variants satisfy this criterion. A cluster is more robustly evidenced when it contains more genetic variants, and particularly if a trait can be found that associates specifically with variants in that cluster. We would advise particular caution in the interpretation of a risk factor as causal on the basis of a small cluster of variants, especially if the majority of variants are in the null cluster.

Our approach in this article was to cluster genetic variants based on their causal estimates for a single risk factor and outcome. There are several advantages to this approach. First, there is a natural interpretation of clusters in terms of the causal effect of the risk factor under investigation. Second, as the causal estimate is the ratio of the genetic association with the outcome to the genetic association with the risk factor, two variants can appear in the same cluster even if one has weaker associations with the risk factor and outcome, and the other has stronger associations. This is important, as the magnitude of genetic associations is independent of the causal pathway by which it influences the risk factor. Third, as cluster assignment is made on the basis of genetic associations with the risk factor and outcome only, genetic associations with other traits can be used to validate cluster membership, and to explore distinct mechanisms by which the risk factor influences the outcome. If data on genetic associations with multiple traits were used to cluster variants, then the clusters might be more precisely defined, but it would not be possible to determine which traits were driving the division into clusters without further analysis. We have previously demonstrated that a group of variants having similar causal estimates for the effect of HDL-cholesterol on CAD risk also had a distinct pattern of associations with blood cell traits, although without using a formal clustering method (Burgess *et al.*, 2020). The associations with blood cell traits suggested a causal pathway relating to platelet aggregation.

To interpret causal estimates as average causal effects, we made parametric assumptions of linearity and homogeneity. We have discussed these assumptions at length previously (Burgess *et al.*, 2016b). Briefly, the associations of genetic variants with traits are typically small, and so while substantial non-linearity is plausible when considering the causal relationship between a risk factor and outcome across the range of the risk factor distribution, it is less likely when considering the impact of small changes in the average level of the risk factor, as estimated in Mendelian randomization. If the homogeneity assumption is not satisfied, then causal estimates can be interpreted as local average causal effects under the assumption of monotonicity. The monotonicity assumption is generally plausible for genetic variants, as it is difficult to conceive a biological reason why a genetic variant would increase the risk factor in one subset of the population, and decrease it in another. This provides another reason why causal estimates from different genetic variants may differ, as the complier populations corresponding to different genetic variants may differ. However, we believe differences in local average causal effects for different complier populations are unlikely to be substantial in practice. A claim that there are multiple causal pathways from the risk factor to the outcome is more plausible when traits can be found that predict cluster membership, particularly if these traits are potential mediators or moderators of the causal effect of the risk factor.

This is not the first method to consider clustering of genetic variants based on their associations with various traits. Previous researchers have considered Bayesian non-negative matrix factorization (Udler *et al.*, 2018), truncated singular value decomposition method (Tanigawa *et al.*, 2019) and hierarchical clustering approaches (Ruth *et al.*, 2020). We believe that our method has important properties when clustering variants based on their causal estimates, which are calculated using associations with a single risk factor and outcome. A potential extension of this framework could consider clustering variants based on associations with multiple traits. There has also been previous work in the Mendelian randomization literature that assigns variants into subgroups. The MRMix method (Qi and Chatterjee, 2019) takes a large number of genetic variants and divides the variants into four subgroups: those associated with the risk factor and outcome through a causal mechanism, those associated with the risk factor and outcome through a pleiotropic mechanism, those associated with the outcome alone and those associated with neither the risk factor nor the outcome. The motivation of the MRMix method is to provide a single estimate of a single causal effect that is robust to some genetic variants not being valid instruments. Our method has a very different objective, which is to find clusters of variants having similar causal effects, rather than to label some variants as valid and others as invalid. Another related method is the MR-TRYX method (Cho *et al.*, 2020). This method also considers whether different genetic variants have similar causal estimates, but instead of focusing on subgroups of variants with similar estimates (as we do here), it instead focuses on individual outliers, and tries to find associations of those variants that may explain why they are outliers. We believe that in most cases, it will not be possible to demonstrate convincingly that a single covariate association explains why a genetic variant is an outlier, and hence our approach, which tries to find groups of variants having similar estimates and then find what is similar between them in terms of their genetic associations, is preferable.

In conclusion, we have proposed a method in the context of Mendelian randomization that clusters genetic variants associated with a given risk factor according to the variant’s associations with the risk factor and outcome. We have shown theoretically and empirically how the method can help elucidate distinct causal pathways by which the risk factor influences the outcome.

## Funding

This work was supported by the UK Medical Research Council (core funding to Stephen Burgess: MC_UU_00002/7 and Paul Kirk: MC_UU_00002/13) and the UK National Institute for Health Research Cambridge Biomedical Research Centre. Stephen Burgess is supported by Sir Henry Dale Fellowship jointly funded by the Wellcome Trust and the Royal Society [204623/Z/16/Z]. The views expressed are those of the authors and not necessarily those of the National Health Service, the National Institute for Health Research or the Department of Health and Social Care.

*Conflict of Interest*: none declared.

## Data availability

All software code and data in this manuscript can be downloaded from https://github.com/cnfoley/mrclust.

## Supplementary Material

btaa778_Supplementary_Data

## References

[btaa778-B1] Angrist J. et al (1996) Identification of causal effects using instrumental variables. J. Am. Stat. Assoc., 91, 444–455.

[btaa778-B2] Baiocchi M. et al (2014) Instrumental variable methods for causal inference. Stat. Med., 33, 2297–2340. 24599889 10.1002/sim.6128 PMC4201653

[btaa778-B3] Burgess S. , Thompson S.G. (2015) Mendelian Randomization: Methods for Using Genetic Variants in Causal Estimation. Chapman & Hall, Boca Raton, FL.

[btaa778-B4] Burgess S. et al (2013) Mendelian randomization analysis with multiple genetic variants using summarized data. Genet. Epidemiol., 37, 658–665. 24114802 10.1002/gepi.21758 PMC4377079

[btaa778-B5] Burgess S. et al (2016a) Bias due to participant overlap in two-sample Mendelian randomization. Genet. Epidemiol., 40, 597–608. 27625185 10.1002/gepi.21998 PMC5082560

[btaa778-B6] Burgess S. et al (2016b) Combining information on multiple instrumental variables in Mendelian randomization: comparison of allele score and summarized data methods. Stat. Med., 35, 1880–1906. 26661904 10.1002/sim.6835 PMC4832315

[btaa778-B7] Burgess S. et al (2017) Dissecting causal pathways using Mendelian randomization with summarized genetic data: application to age at menarche and risk of breast cancer. Genetics, 207, 481–487. 28835472 10.1534/genetics.117.300191 PMC5629317

[btaa778-B8] Burgess S. et al (2020) A robust and efficient method for Mendelian randomization with hundreds of genetic variants. Nat. Commun., 11, 376. 31953392 10.1038/s41467-019-14156-4 PMC6969055

[btaa778-B9] Cho Y. et al (2020) Exploiting horizontal pleiotropy to search for causal pathways within a Mendelian randomization framework. Nat. Commun., 11, 1010. 32081875 10.1038/s41467-020-14452-4 PMC7035387

[btaa778-B10] Clarke P.S. , Windmeijer F. (2012) Instrumental variable estimators for binary outcomes. J. Am. Stat. Assoc., 107, 1638–1652.

[btaa778-B11] Crook O.M. et al (2018) A Bayesian mixture modelling approach for spatial proteomics. PLoS Comput. Biol., 14, e1006516. 30481170 10.1371/journal.pcbi.1006516 PMC6258510

[btaa778-B12] Davey Smith G. , Ebrahim S. (2003) ‘ Mendelian randomization’: can genetic epidemiology contribute to understanding environmental determinants of disease? Int. J. Epidemiol., 32, 1–22. 12689998 10.1093/ije/dyg070

[btaa778-B13] Didelez V. , Sheehan N. (2007) Mendelian randomization as an instrumental variable approach to causal inference. Stat. Methods Med. Res., 16, 309–330. 17715159 10.1177/0962280206077743

[btaa778-B14] Didelez V. et al (2010) Assumptions of IV methods for observational epidemiology. Stat. Sci., 25, 22–40.

[btaa778-B15] Evangelou E. et al; the Million Veteran Program. (2018) Genetic analysis of over one million people identifies 535 novel loci for blood pressure. Nat. Genet., 50, 1412–1425. 30224653 10.1038/s41588-018-0205-x PMC6284793

[btaa778-B16] Greenland S. (2000) An introduction to instrumental variables for epidemiologists. Int. J. Epidemiol., 29, 722–729. 10922351 10.1093/ije/29.4.722

[btaa778-B17] Hernán M.A. , Robins J.M. (2006) Instruments for causal inference: an epidemiologist’s dream? Epidemiology, 17, 360–372. 16755261 10.1097/01.ede.0000222409.00878.37

[btaa778-B18] Imbens G.W. , Angrist J.D. (1994) Identification and estimation of local average treatment effects. Econometrica, 62, 467–475.

[btaa778-B19] Johnson T. (2013) *gtx: Genetics ToolboX.* R package version 0.0.8. https://cran.r-project.org/src/contrib/Archive/gtx/.

[btaa778-B20] Lawlor D. et al (2008) Mendelian randomization: using genes as instruments for making causal inferences in epidemiology. Stat. Med., 27, 1133–1163. 17886233 10.1002/sim.3034

[btaa778-B21] Pearl J. (2000) Causality: Models, Reasoning, and Inference. Cambridge University Press, New York, NY, USA.

[btaa778-B22] Plenge R. et al (2013) Validating therapeutic targets through human genetics. Nat. Rev. Drug Disc., 12, 581–594. 10.1038/nrd4051 23868113

[btaa778-B23] Qi G. , Chatterjee N. (2019) Mendelian randomization analysis using mixture models for robust and efficient estimation of causal effects. Nat. Commun., 10, 1941. 31028273 10.1038/s41467-019-09432-2 PMC6486646

[btaa778-B24] Rand W.M. (1971) Objective criteria for the evaluation of clustering methods. J. Am. Stat. Assoc., 66, 846–850.

[btaa778-B25] Ruth K.S. et al; The Endometrial Cancer Association Consortium. (2020) Using human genetics to understand the disease impacts of testosterone in men and women. Nat. Med., 26, 252–258. 32042192 10.1038/s41591-020-0751-5 PMC7025895

[btaa778-B26] Scrucca L. et al (2016) mclust 5: clustering, classification and density estimation using Gaussian finite mixture models. R. J., 8, 289–317. 27818791 PMC5096736

[btaa778-B27] Staley J.R. et al (2016) PhenoScanner: a database of human genotype-phenotype associations. Bioinformatics, 32, 3207–3209. 27318201 10.1093/bioinformatics/btw373 PMC5048068

[btaa778-B28] Swanson S. , Hernán M. (2013) Commentary: how to report instrumental variable analyses (suggestions welcome). Epidemiology, 24, 370–374. 23549180 10.1097/EDE.0b013e31828d0590

[btaa778-B29] Tanigawa Y. et al (2019) Components of genetic associations across 2,138 phenotypes in the UK Biobank highlight adipocyte biology. Nat. Commun., 10, 4064. 31492854 10.1038/s41467-019-11953-9 PMC6731283

[btaa778-B30] The International Consortium for Blood Pressure Genome-Wide Association Studies. (2011) Genetic variants in novel pathways influence blood pressure and cardiovascular disease risk. Nature, 478, 103–109. 21909115 10.1038/nature10405 PMC3340926

[btaa778-B31] Udler M.S. et al; on behalf of METASTROKE and the ISGC. (2018) Type 2 diabetes genetic loci informed by multi-trait associations point to disease mechanisms and subtypes: a soft clustering analysis. PLoS Med., 15, e1002654. 30240442 10.1371/journal.pmed.1002654 PMC6150463

[btaa778-B32] van der Harst P. , Verweij N. (2018) Identification of 64 novel genetic loci provides an expanded view on the genetic architecture of coronary artery disease. Circ. Res., 122, 433–443. 29212778 10.1161/CIRCRESAHA.117.312086 PMC5805277

[btaa778-B33] Verbanck M. et al (2018) Detection of widespread horizontal pleiotropy in causal relationships inferred from Mendelian randomization between complex traits and diseases. Nat. Genet., 50, 693–698. 29686387 10.1038/s41588-018-0099-7 PMC6083837

[btaa778-B34] Voight B. et al (2012) Plasma HDL cholesterol and risk of myocardial infarction: a mendelian randomisation study. Lancet, 380, 572–580. 22607825 10.1016/S0140-6736(12)60312-2 PMC3419820

[btaa778-B35] Walter S. et al (2015) Revisiting mendelian randomization studies of the effect of body mass index on depression. Am. J. Med. Genet. B Neuropsychiatric Genet., 168, 108–115. 10.1002/ajmg.b.32286 PMC4387873 25656382

[btaa778-B36] Wooldridge J. (2009) Introductory econometrics: A modern approach. Chapter 15: Instrumental Variables Estimation and Two Stage Least Squares. South-Western, Nashville, TN.

