# Supplementary material for: MR-Clust: clustering of genetic variants in Mendelian randomization with similar causal estimates
Source: Bioinformatics. 2020 Sep 11;37(4):531–41. doi: 10.1093/bioinformatics/btaa778 (PMC8088327; doi:10.1093/bioinformatics/btaa778)
Supplement: btaa778_Supplementary_Data [file btaa778_supplementary_data.pdf]

Appendix to “MR-Clust: Clustering of genetic variants in Mendelian randomization with similar causal estimates”

Christopher N Foley Amy M Mason Paul D W Kirk
Stephen Burgess

A Clustered heterogeneity under linearity and homogeneity assumptions

We consider the scenario in which there are linear and homogeneous relationships between genetic variants $G_1, \dots, G_J$, a risk factor X, an outcome Y, and (initially) a single risk factor–outcome confounder U. We assume the following linear structural models:

$$\begin{aligned} X &= \boldsymbol{\beta}^T \boldsymbol{G} + \phi U + \epsilon_X, & \epsilon_X &\perp \{\boldsymbol{G}, U\}, \\ U &= \boldsymbol{\eta}^T \boldsymbol{G} + \epsilon_U, & \epsilon_U &\perp \boldsymbol{G}, \\ Y &= \boldsymbol{\delta}^T \boldsymbol{G} + \theta X + \xi U + \epsilon_Y, & \epsilon_Y &\perp \{\boldsymbol{G}, X, U\}, \end{aligned} \tag{A1}$$

where bold face represents a vector and the epsilon terms represent error in each variable. We assume the ϕ and ξ are non-zero, and consider ratio estimates for genetic variants with different values of β_j, η_j and δ_j. The expected value of the ratio estimate (the ratio estimand) using the jth variant is:

$$\lim_{n \to \infty} \hat{\theta}_j = \theta + \frac{\delta_j + \xi \eta_j}{\beta_j + \phi \eta_j} \tag{A2}$$

We consider different values of β_j, η_j and δ_j, and the resulting ratio estimands. There are eight possible scenarios for these parameters equalling zero or differing from zero:

β_j	η_j	δ_j	Ratio estimand	Constant function of β_j, η_j, and δ_j?
$= 0$	$= 0$	$= 0$	Undefined	NA
$\neq 0$	$= 0$	$= 0$	θ	Yes
$= 0$	$\neq 0$	$= 0$	$\theta + \frac{\xi}{\phi}$	Yes
$= 0$	$= 0$	$\neq 0$	Undefined	NA
$\neq 0$	$\neq 0$	$= 0$	$\theta + \frac{\xi \eta_j}{\beta_j + \phi \eta_j}$	No
$\neq 0$	$= 0$	$\neq 0$	$\theta + \frac{\delta_j}{\beta_j}$	No
$= 0$	$\neq 0$	$\neq 0$	$\theta + \frac{\delta_j + \xi \eta_j}{\phi \eta_j}$	No
$\neq 0$	$\neq 0$	$\neq 0$	$\theta + \frac{\delta_j + \xi \eta_j}{\beta_j + \phi \eta_j}$	No

Table A1: Exhaustive representation of combinations of possible causal effects of genetic variant j on the risk factor (present if $\beta_j \neq 0$), confounder (present if $\eta_j \neq 0$), and outcome (present if $\delta_j \neq 0$). Only two possibilities lead to the ratio estimand being a constant function of β_j, η_j, and δ_j.

The two situations in which ratio estimands take the same values for multiple genetic variants with different values of β_j, η_j and δ_j are:

1. Genetic variants influence risk factor only ($\beta_j \neq 0$, $\eta_j = 0$, $\delta_j = 0$), and
2. Genetic variants influence confounder only ($\beta_j = 0$, $\eta_j \neq 0$, $\delta_j = 0$).

While it is possible for ratio estimands to exactly coincide for other values of β_j, η_j and δ_j due to chance, this is vanishingly unlikely.

If we generalize further to a scenario with multiple risk factor–outcome confounders, similar considerations show that ratio estimands will coincide exactly when the genetic variants influence the risk factor only, or one confounder only. This suggests that clustered heterogeneity in the linear and homogeneous scenario corresponds to the situation where genetic

variants influence the outcome via a single causal mediator: either the nominated risk factor, or a confounder of the risk factor and outcome. In both situations, there is a common causal pathway from variants in the cluster to the outcome. This corresponds to the diagram of Figure 2.

B Specification of the junk distribution

To ensure that the distribution of ratio estimates in the junk cluster is near constant across the range of observations from a given sample, whilst also accounting for uncertainty in the ratio estimates $\hat{\theta}_j$ via their standard errors $\hat{\sigma}_j$, we set the scale parameter ψ in the generalised t-distribution for the junk cluster to

$$\hat{\psi} = \mid \hat{\theta}_{\text{max}} - \hat{\theta}_{\text{min}} \mid + 2\hat{\sigma}_{\text{max}},$$
$$\mathcal{T}\left(\nu, \mu, \psi\right) \approx \frac{\Gamma\left(\frac{\nu+1}{2}\right)}{\Gamma\left(\nu/2\right)\sqrt{\pi\nu}\psi},$$

in our applications, where $\hat{\theta}_{\text{max}}$ is the maximum of the ratio estimates, $\hat{\theta}_{\text{min}}$ is the minimum of the ratio estimates, and $\hat{\sigma}_{\text{max}}$ is the maximum of the standard errors of the ratio estimates. This means that the density of the junk cluster will vary between samples, automatically accounting for differences in the range and precision of the ratio estimates. The junk cluster density is a proper density that is approximately uniform across the range of plausible values of the ratio estimates.

C Testing for associations with variants in a given cluster

As part of the exploratory *post hoc* investigation of whether genetic variants in a cluster associate preferentially with a given trait, we perform a statistical test. We compare the number of variants in the cluster that associate with the trait at a given p-value threshold (n_{11}), the number of variants in the cluster that do not associate with the trait at a given p-value threshold (n_{12}), the number of variants not in the cluster that associate with the trait at a given p-value threshold (n_{21}), and the number of variants not in the cluster that do not associate with the trait at a given p-value threshold (n_{22}). We consider the number of ways in which n_{11} associations are observed for $n_{11}+n_{12}$ variants as a proportion of the number of ways that $n_{11}+n_{21}$ associations could arise for n total variants (where $n = n_{11}+n_{12}+n_{21}+n_{22}$). This is given by the hypergeometric distribution as:

$$\frac{\binom{n_{11}+n_{21}}{n_{11}}\binom{n_{12}+n_{22}}{n_{12}}}{\binom{n}{n_{11}+n_{12}}}. \tag{A3}$$

The relevant p-value is given by summing up these probabilities for n_{11} traits associating with variants in the cluster and n_{21} associating with variants not in the cluster, $n_{11}+1$ traits associating with variants in the cluster and $n_{21}-1$ associating with variants not in the cluster, and so on up to $n_{11}+n_{21}$ traits associating with variants in the cluster and zero associating with variants not in the cluster.

Table A2: Summary of genetic variants and assignment to clusters in separate analyses for systolic blood pressure (SBP), diastolic blood pressure (DBP), and pulse pressure (PP): cluster number (based on highest conditional probability), cluster mean, ratio estimate (Estimate) for that variant, and its standard error (SE).

	SBP				DBP				PP			
rsid	Cluster	Mean	Est	SE	Cluster	Mean	Est	SE	Cluster	Mean	Est	SE
rs17249754	1	-0.071	-0.069	0.009	1	-0.128	-0.136	0.018	-	-	-0.143	0.019
rs1438896	1	-0.071	-0.090	0.025	1	-0.128	-0.107	0.029	-	-	-	-
rs4494250	1	-0.071	-0.058	0.023	-	-	-0.084	0.034	-	-	-	-
rs35410524	1	-0.071	-0.105	0.024	-	-	-	-	-	-	-	-
rs3184504	4	0.113	0.100	0.010	4	0.131	0.131	0.012	-	-	0.390	0.037
rs12579720	4	0.113	0.119	0.021	4	0.131	0.132	0.024	-	-	-	-
rs12940887	4	0.113	0.140	0.020	4	0.131	0.150	0.022	-	-	-	-
rs6797587	4	0.113	0.124	0.019	4	0.131	0.157	0.024	-	-	-	-
rs2521501	4	0.113	0.118	0.011	3	0.204	0.186	0.018	4	0.231	0.273	0.026
rs1063281	4	0.113	0.137	0.025	3	0.204	0.237	0.043	-	-	-	-
rs112557609	4	0.113	0.121	0.025	-	-	-	-	-	-	0.180	0.037
rs2972146	4	0.113	0.113	0.022	-	-	0.202	0.039	-	-	-	-
rs11128722	4	0.113	0.121	0.021	-	-	-	-	-	-	-	-
rs2782980	5	0.053	0.058	0.017	2	0.077	0.082	0.024	-	-	-	-
rs1813353	5	0.053	0.049	0.014	2	0.077	0.076	0.022	-	-	-	-
rs1530440	5	0.053	0.049	0.013	2	0.077	0.068	0.018	-	-	-	-
rs880315	5	0.053	0.050	0.012	2	0.077	0.103	0.024	2	0.122	0.104	0.024
rs17367504	5	0.053	0.046	0.009	2	0.077	0.076	0.015	2	0.122	0.125	0.024
rs751984	5	0.053	0.070	0.017	2	0.077	0.084	0.020	-	-	-	-
rs1378942	5	0.053	0.049	0.011	2	0.077	0.064	0.015	-	-	-	-
rs12921187	5	0.053	0.060	0.024	2	0.077	0.094	0.037	-	-	-	-
rs2240736	5	0.053	0.057	0.015	2	0.077	0.099	0.025	2	0.122	0.132	0.034
rs4308	5	0.053	0.042	0.022	2	0.077	0.062	0.032	-	-	-	-
rs167479	5	0.053	0.081	0.015	2	0.077	0.101	0.018	-	-	-	-
rs1327235	5	0.053	0.060	0.016	2	0.077	0.089	0.023	-	-	-	-
rs6015450	5	0.053	0.058	0.012	2	0.077	0.079	0.017	-	-	-	-
rs12627651	5	0.053	0.039	0.016	2	0.077	0.065	0.026	-	-	0.095	0.038
rs1446468	5	0.053	0.041	0.012	2	0.077	0.076	0.022	-	-	0.094	0.027
rs76326501	5	0.053	0.043	0.019	2	0.077	0.066	0.029	-	-	-	-
rs13082711	5	0.053	0.044	0.022	2	0.077	0.067	0.033	-	-	-	-
rs1458038	5	0.053	0.058	0.008	2	0.077	0.093	0.014	2	0.122	0.146	0.021
rs1173771	5	0.053	0.036	0.010	2	0.077	0.073	0.020	3	0.038	0.068	0.019
rs17080102	5	0.053	0.063	0.014	2	0.077	0.089	0.021	-	-	-	-
rs6557876	5	0.053	0.039	0.018	2	0.077	0.073	0.033	-	-	-	-
rs11191548	5	0.053	0.063	0.008	4	0.131	0.141	0.019	2	0.122	0.123	0.017
rs17608766	5	0.053	0.076	0.012	3	0.204	0.248	0.040	2	0.122	0.105	0.017
rs633185	5	0.053	0.068	0.011	-	-	0.121	0.020	2	0.122	0.162	0.027
rs7129220	5	0.053	0.069	0.021	-	-	0.143	0.045	-	-	-	-
rs381815	5	0.053	0.074	0.016	-	-	0.131	0.029	-	-	-	-
rs3820068	5	0.053	0.046	0.021	-	-	-	-	-	-	0.079	0.037
rs11229457	5	0.053	0.062	0.024	-	-	0.113	0.043	-	-	-	-
rs2076328	5	0.053	0.061	0.021	-	-	-	-	-	-	0.101	0.035
rs10850411	5	0.053	0.070	0.018	-	-	0.115	0.030	-	-	-	-
rs2004776	5	0.053	0.074	0.019	-	-	0.105	0.027	-	-	-	-
rs9323988	5	0.053	0.047	0.024	-	-	-	-	-	-	0.077	0.040
rs1036477	5	0.053	0.054	0.026	-	-	-	-	3	0.038	0.047	0.022
rs17638167	5	0.053	0.069	0.023	-	-	-	-	-	-	-	-
rs6060114	5	0.053	0.053	0.028	-	-	-	-	-	-	-	-
rs13420463	5	0.053	0.049	0.023	-	-	-	-	-	-	-	-
rs347591	5	0.053	0.065	0.021	-	-	0.125	0.040	-	-	-	-
rs6783086	5	0.053	0.058	0.023	-	-	0.102	0.040	-	-	-	-
rs2291435	5	0.053	0.061	0.021	-	-	0.109	0.037	-	-	-	-
rs871606	5	0.053	0.053	0.021	-	-	-	-	3	0.038	0.048	0.019
rs13359291	5	0.053	0.070	0.019	-	-	-	-	2	0.122	0.120	0.032
rs1563788	5	0.053	0.062	0.020	-	-	-	-	-	-	0.103	0.033
rs17477177	5	0.053	0.048	0.012	-	-	-	-	-	-	-	-
rs10224002	5	0.053	0.057	0.027	-	-	-	-	-	-	-	-
rs111245230	5	0.053	0.085	0.023	-	-	0.146	0.040	-	-	-	-
rs13139571	6	0.208	0.201	0.022	3	0.204	0.258	0.029	-	-	-	-
rs3918226	6	0.208	0.196	0.021	3	0.204	0.196	0.021	-	-	-	-
rs11556924	6	0.208	0.220	0.022	-	-	0.386	0.039	-	-	-	-
rs2107595	6	0.208	0.249	0.024	-	-	-	-	4	0.231	0.229	0.022
rs12906962	-	-	0.039	0.023	2	0.077	0.055	0.032	-	-	-	-
rs13112725	-	-	0.034	0.016	2	0.077	0.063	0.030	-	-	0.078	0.038
rs2969070	-	-	0.038	0.019	2	0.077	0.053	0.027	-	-	-	-

(continued from previous page)

rsid	SBP				DBP				PP			
	Cluster	Mean	Estimate	SE	Cluster	Mean	Estimate	SE	Cluster	Mean	Estimate	SE
rs7777128	-	-	0.035	0.019	2	0.077	0.058	0.032	-	-	-	-
rs12405515	-	-	-	-	2	0.077	0.087	0.035	-	-	-	-
rs9827472	-	-	-	-	2	0.077	0.092	0.041	-	-	-	-
rs12521868	-	-	-	-	2	0.077	0.061	0.036	-	-	-	-
rs9687065	-	-	-	-	2	0.077	0.092	0.040	-	-	-	-
rs6271	-	-	-	-	2	0.077	0.098	0.031	-	-	-	-
rs4387287	-	-	-	-	1	-0.128	-0.197	0.045	-	-	-	-
rs11030119	-	-	-	-	1	-0.128	-0.123	0.041	-	-	-	-
rs2304130	-	-	-	-	1	-0.128	-0.119	0.042	-	-	-	-
rs62104477	-	-	-	-	1	-0.128	-0.126	0.047	-	-	-	-
rs918466	-	-	-	-	1	-0.128	-0.101	0.032	-	-	-	-
rs687621	-	-	-	-	1	-0.128	-0.203	0.040	-	-	-	-
rs3741378	-	-	0.096	0.018	3	0.204	0.219	0.041	-	-	-	-
rs9815354	-	-	-	-	-	-	-0.050	0.023	3	0.038	0.045	0.021
rs34872471	-	-	-	-	-	-	-	-	2	0.122	0.144	0.039
rs60199046	-	-	-	-	-	-	-	-	2	0.122	0.105	0.028
rs12628032	-	-	-	-	-	-	-	-	2	0.122	0.132	0.032
rs869396	-	-	-	-	-	-	-	-	2	0.122	0.158	0.031
rs917275	-	-	-	-	-	-	-	-	2	0.122	0.138	0.033
rs35261357	-	-	0.166	0.021	-	-	-	-	4	0.231	0.227	0.029
rs9337951	-	-	-	-	-	-	-	-	4	0.231	0.264	0.031
rs9549328	-	-	-	-	-	-	-	-	4	0.231	0.216	0.040
rs7500448	-	-	-	-	-	-	-	-	4	0.231	0.202	0.025
rs112184198	-	-	-0.028	0.018	-	-	-0.050	0.032	-	-	-0.059	0.038
rs4373814	-	-	0.017	0.023	-	-	0.027	0.036	-	-	-	-
rs932764	-	-	0.004	0.014	-	-	0.011	0.034	-	-	0.007	0.023
rs17030613	-	-	-0.015	0.018	-	-	-0.025	0.029	-	-	-	-
rs2932538	-	-	0.005	0.017	-	-	0.007	0.025	-	-	-	-
rs4757391	-	-	0.021	0.014	-	-	0.035	0.023	-	-	0.054	0.036
rs5219	-	-	0.012	0.018	-	-	0.023	0.037	-	-	0.025	0.039
rs661348	-	-	0.022	0.018	-	-	0.047	0.037	-	-	0.039	0.031
rs7103648	-	-	-0.013	0.017	-	-	-0.022	0.028	-	-	-	-
rs35444	-	-	0.027	0.017	-	-	0.041	0.026	-	-	-	-
rs12408022	-	-	0.035	0.026	-	-	0.060	0.044	-	-	-	-
rs7302981	-	-	-0.012	0.014	-	-	-0.019	0.023	-	-	-0.028	0.034
rs73099903	-	-	0.034	0.024	-	-	-	-	-	-	-	-
rs7297416	-	-	0.040	0.022	-	-	-	-	-	-	0.065	0.036
rs9508495	-	-	0.004	0.017	-	-	0.007	0.030	-	-	0.010	0.040
rs7515635	-	-	0.024	0.026	-	-	-	-	-	-	-	-
rs8904	-	-	0.014	0.018	-	-	-	-	-	-	0.021	0.027
rs9888615	-	-	0.045	0.026	-	-	-	-	-	-	0.064	0.038
rs2759308	-	-	0.041	0.022	-	-	-	-	-	-	0.067	0.037
rs13333226	-	-	0.017	0.019	-	-	0.023	0.025	-	-	-	-
rs12946454	-	-	0.015	0.018	-	-	0.033	0.039	-	-	0.027	0.032
rs7406910	-	-	0.018	0.019	-	-	-	-	-	-	0.027	0.029
rs12958173	-	-	0.001	0.016	-	-	0.002	0.033	-	-	0.002	0.034
rs7236548	-	-	0.025	0.026	-	-	-	-	-	-	0.027	0.029
rs4247374	-	-	0.025	0.017	-	-	0.037	0.026	-	-	-	-
rs6031435	-	-	0.031	0.025	-	-	-	-	-	-	0.050	0.041
rs16823124	-	-	0.105	0.026	-	-	0.135	0.034	-	-	-	-
rs7592578	-	-	0.006	0.021	-	-	0.011	0.039	-	-	-	-
rs55780018	-	-	0.015	0.018	-	-	0.034	0.041	-	-	0.026	0.031
rs1275988	-	-	0.016	0.011	-	-	0.030	0.021	-	-	0.034	0.024
rs1975487	-	-	0.031	0.024	-	-	0.040	0.030	-	-	-	-
rs16851397	-	-	-0.002	0.021	-	-	-0.003	0.030	-	-	-	-
rs419076	-	-	0.015	0.014	-	-	0.024	0.022	-	-	0.042	0.040
rs13107325	-	-	0.000	0.013	-	-	0.000	0.019	-	-	0.001	0.036
rs2014912	-	-	0.020	0.015	-	-	-	-	-	-	0.032	0.024
rs10077885	-	-	0.007	0.021	-	-	0.013	0.039	-	-	-	-
rs6891344	-	-	0.033	0.024	-	-	0.044	0.031	-	-	-	-
rs6595838	-	-	0.098	0.026	-	-	-	-	-	-	-	-
rs13209747	-	-	0.000	0.014	-	-	0.000	0.022	-	-	0.000	0.032
rs9349379	-	-	-0.409	0.022	-	-	-	-	-	-	-0.566	0.031
rs1799945	-	-	-0.024	0.014	-	-	-0.035	0.020	-	-	-	-
rs409558	-	-	-0.010	0.018	-	-	-	-	-	-	-0.014	0.026
rs4728142	-	-	-0.012	0.026	-	-	-	-	-	-	-0.017	0.040
rs891511	-	-	0.086	0.025	-	-	0.103	0.029	-	-	-	-
rs6969780	-	-	0.032	0.026	-	-	-	-	-	-	-	-
rs76206723	-	-	0.035	0.026	-	-	-	-	-	-	0.036	0.026
rs11977526	-	-	-0.050	0.021	-	-	-	-	-	-	-0.040	0.017

(continued from previous page)

rsid	SBP				DBP				PP			
	Cluster	Mean	Estimate	SE	Cluster	Mean	Estimate	SE	Cluster	Mean	Estimate	SE
rs35783704	-	-	0.021	0.019	-	-	0.045	0.041	-	-	0.042	0.039
rs2898290	-	-	0.001	0.016	-	-	0.002	0.040	-	-	0.002	0.027
rs4454254	-	-	0.006	0.026	-	-	-	-	-	-	0.006	0.027
rs34591516	-	-	0.033	0.024	-	-	0.065	0.048	-	-	-	-
rs1449544	-	-	0.031	0.025	-	-	-	-	-	-	0.039	0.032
rs72765298	-	-	-0.020	0.026	-	-	-	-	-	-	-0.024	0.030
rs4245739	-	-	-	-	-	-	0.092	0.042	-	-	-	-
rs1060105	-	-	-	-	-	-	0.024	0.043	-	-	-	-
rs6429422	-	-	-	-	-	-	-0.018	0.031	-	-	-	-
rs7178615	-	-	-	-	-	-	0.042	0.044	-	-	-	-
rs72799341	-	-	-	-	-	-	-0.038	0.042	-	-	-	-
rs1126464	-	-	-	-	-	-	-0.037	0.031	-	-	-	-
rs78378222	-	-	-	-	-	-	-0.017	0.046	-	-	0.014	0.038
rs745821	-	-	-	-	-	-	0.092	0.045	-	-	-	-
rs6095241	-	-	-	-	-	-	-0.082	0.045	-	-	-	-
rs6108168	-	-	-	-	-	-	0.122	0.045	-	-	-	-
rs9306160	-	-	-	-	-	-	0.151	0.034	-	-	-	-
rs79146658	-	-	-	-	-	-	0.034	0.038	-	-	-0.037	0.041
rs4952611	-	-	-	-	-	-	-0.016	0.044	-	-	-	-
rs2579519	-	-	-	-	-	-	-0.011	0.039	-	-	0.011	0.041
rs2306374	-	-	-	-	-	-	0.349	0.042	-	-	-	-
rs12374077	-	-	-	-	-	-	-0.009	0.046	-	-	-	-
rs9810888	-	-	-	-	-	-	0.038	0.045	-	-	-	-
rs6825911	-	-	-	-	-	-	0.107	0.039	-	-	-	-
rs66887589	-	-	-	-	-	-	0.208	0.045	-	-	-	-
rs73030266	-	-	-	-	-	-	0.151	0.038	-	-	-	-
rs10943605	-	-	-	-	-	-	0.113	0.036	-	-	-	-
rs2071518	-	-	-	-	-	-	-0.038	0.045	-	-	0.015	0.018
rs76452347	-	-	-	-	-	-	-0.017	0.046	-	-	-	-
rs2289125	-	-	-	-	-	-	-	-	-	-	-0.026	0.023
rs2761436	-	-	-	-	-	-	-	-	-	-	0.047	0.034
rs452036	-	-	-	-	-	-	-	-	-	-	0.000	0.027
rs33063	-	-	-	-	-	-	-	-	-	-	-0.038	0.034
rs2645466	-	-	-	-	-	-	-	-	-	-	0.055	0.035
rs740698	-	-	-	-	-	-	-	-	-	-	0.047	0.039
rs28427409	-	-	-	-	-	-	-	-	-	-	0.058	0.034
rs36010659	-	-	-	-	-	-	-	-	-	-	0.021	0.039
rs9662255	-	-	-	-	-	-	-	-	-	-	0.041	0.038
rs6081613	-	-	-	-	-	-	-	-	-	-	-0.046	0.030
rs7255	-	-	-	-	-	-	-	-	-	-	-0.074	0.033
rs9479200	-	-	-	-	-	-	-	-	-	-	0.014	0.031
rs449789	-	-	-	-	-	-	-	-	-	-	0.180	0.036
rs1322639	-	-	-	-	-	-	-	-	-	-	0.004	0.035
rs1953126	-	-	-	-	-	-	-	-	-	-	0.059	0.037
rs10818775	-	-	-	-	-	-	-	-	-	-	0.031	0.033

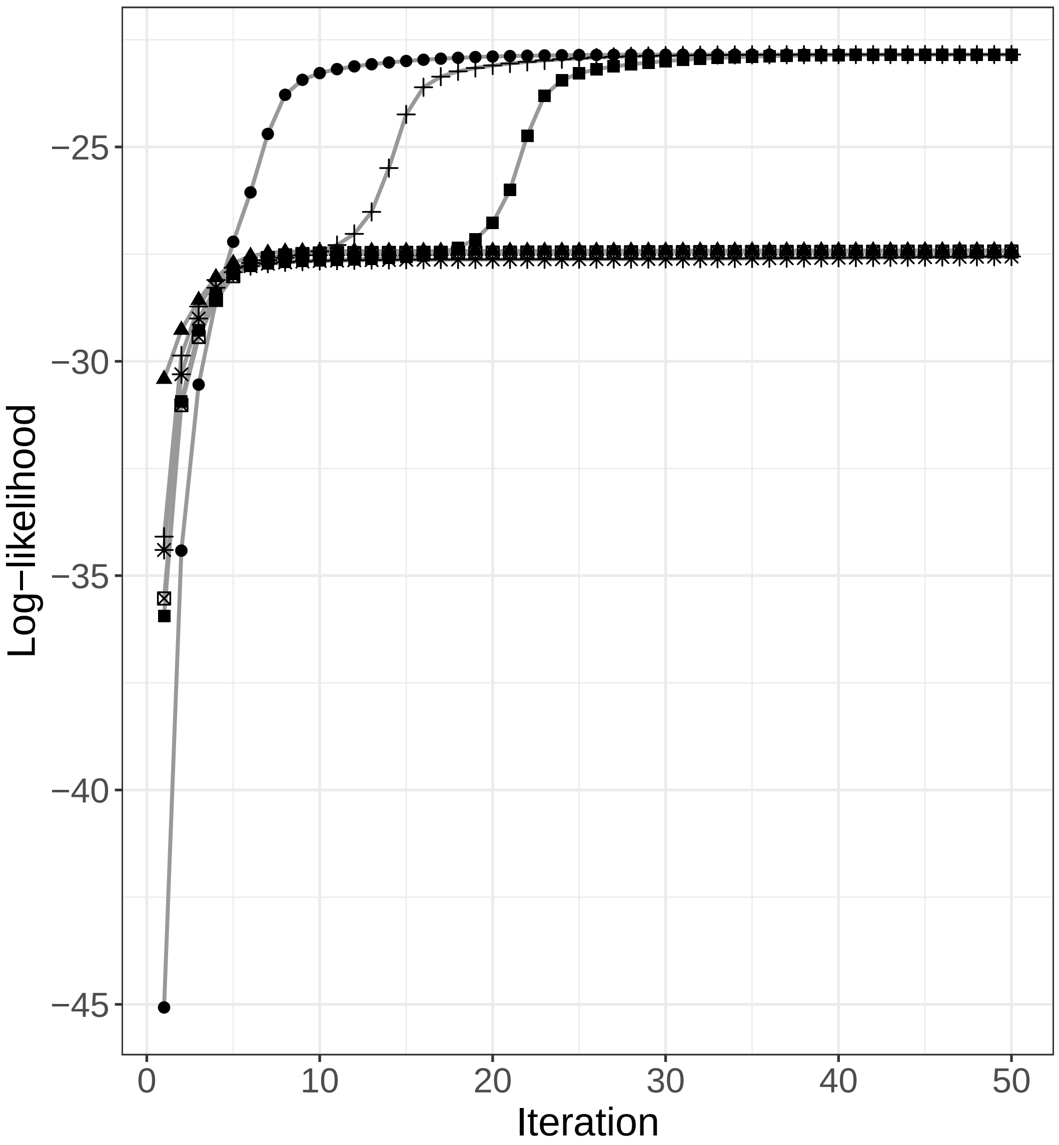

Figure A1: Convergence to the maximum likelihood estimate across iterations for six initializations for a specifically-chosen dataset from scenario 4 that demonstrates failure to converge to the same estimates for all initializations. In our experience, this was not common, but convergence should be assessed carefully in practice.

Clustering results for applied example using MR-Clust, TAGM, and Mclust methods.

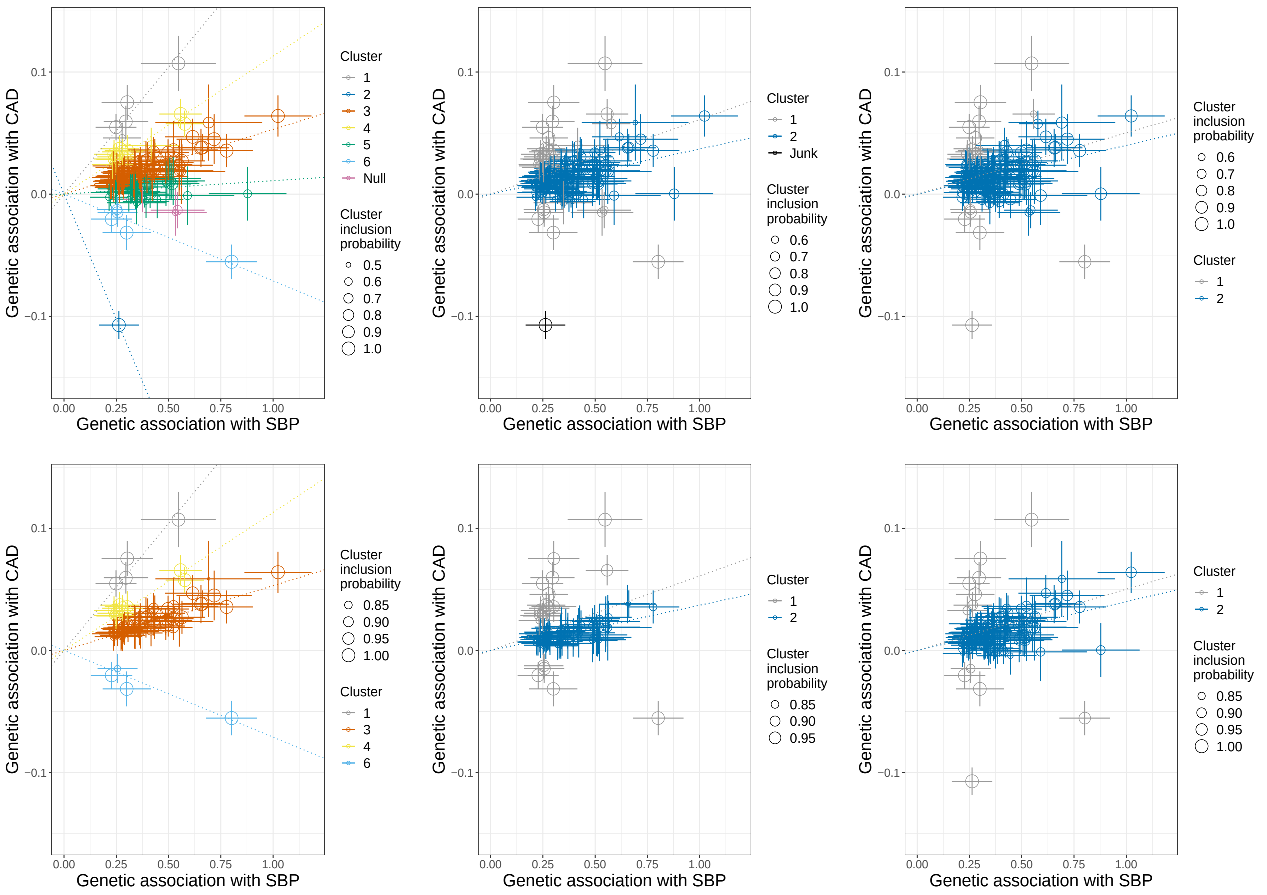

Figure A2: Clusters from genetic associations with systolic blood pressure (mmHg) and coronary artery disease risk (log odds) per additional blood pressure-increasing allele using MR-Clust (left), TAGM (centre) and Mclust (right). Top row: method version (A) – each variant is assigned to the cluster with the greatest conditional probability. Bottom row: method version (B) – variants are only assigned to a cluster if the conditional probability is ≥ 0.8, and clusters are only displayed if at least 4 variants are assigned to the cluster. In version (B), MR-Clust identified 4 substantive clusters consisting of observations whose magnitudes of effect as well as directions are similar. In contrast, TAGM and Mclust identified 2 clusters with each method clustering observations with different directions of effect.

Genetic variant	Effect allele	Genetic association with... SBP	DBP	PP	CAD risk	Trunk fat %	Impedance of arm	Arm fat %
rs17249754	G	0.802 (0.062)	0.408 (0.037)	0.388 (0.042)	-0.055 (0.007)	-0.005 (0.003)	0.002 (0.002)	-0.005 (0.002)
rs1438896	T	0.228 (0.050)	0.191 (0.029)	0.045 (0.034)	-0.020 (0.006)	0.003 (0.002)	-0.004 (0.002)	0.001 (0.002)
rs4494250	A	0.256 (0.049)	0.177 (0.029)	0.062 (0.033)	-0.015 (0.006)	0.008 (0.002)	0.010 (0.002)	0.004 (0.002)
rs35410524	T	0.300 (0.059)	0.141 (0.035)	0.162 (0.040)	-0.032 (0.007)	0.003 (0.003)	-0.004 (0.002)	0.003 (0.002)
rs11030119	G	0.170 (0.051)	0.133 (0.030)	0.053 (0.034)	-0.016 (0.006)	-0.023 (0.002)	0.013 (0.002)	-0.022 (0.002)
rs2304130	G	0.307 (0.086)	0.234 (0.051)	0.067 (0.058)	-0.028 (0.010)	-0.020 (0.004)	-0.007 (0.003)	-0.014 (0.003)
rs62104477	T	0.087 (0.049)	0.130 (0.029)	-0.026 (0.033)	-0.016 (0.006)	0.018 (0.002)	-0.007 (0.002)	0.016 (0.002)
rs918466	G	0.139 (0.048)	0.164 (0.028)	-0.042 (0.032)	-0.017 (0.005)	-0.010 (0.002)	-0.009 (0.002)	-0.006 (0.002)
rs687621	A	0.098 (0.048)	0.133 (0.029)	-0.026 (0.033)	-0.027 (0.005)	-0.013 (0.002)	-0.015 (0.002)	-0.013 (0.002)
rs4387287	A	0.195 (0.062)	0.167 (0.037)	0.020 (0.042)	-0.033 (0.008)	-	-	-

Table A3: Genetic associations (beta-coefficients and standard errors) for variants in the cluster with negative causal effect of blood pressure on coronary artery disease risk. Genetic associations with blood pressure (mmHg) were estimated in 299 024 participants of European ancestry from the International Consortium for Blood Pressure. Genetic associations with coronary artery disease (CAD) risk (log odds ratios) were estimated in 122 733 cases and 424 528 controls primarily of European descent from the CARDIoGRAMplusC4D consortium and UK Biobank. Genetic associations with the adiposity traits (SD units) were estimated in 337 199 participants of European descent from UK Biobank (Ben Neale estimates). Associations in UK Biobank for rs4387287 were not available.

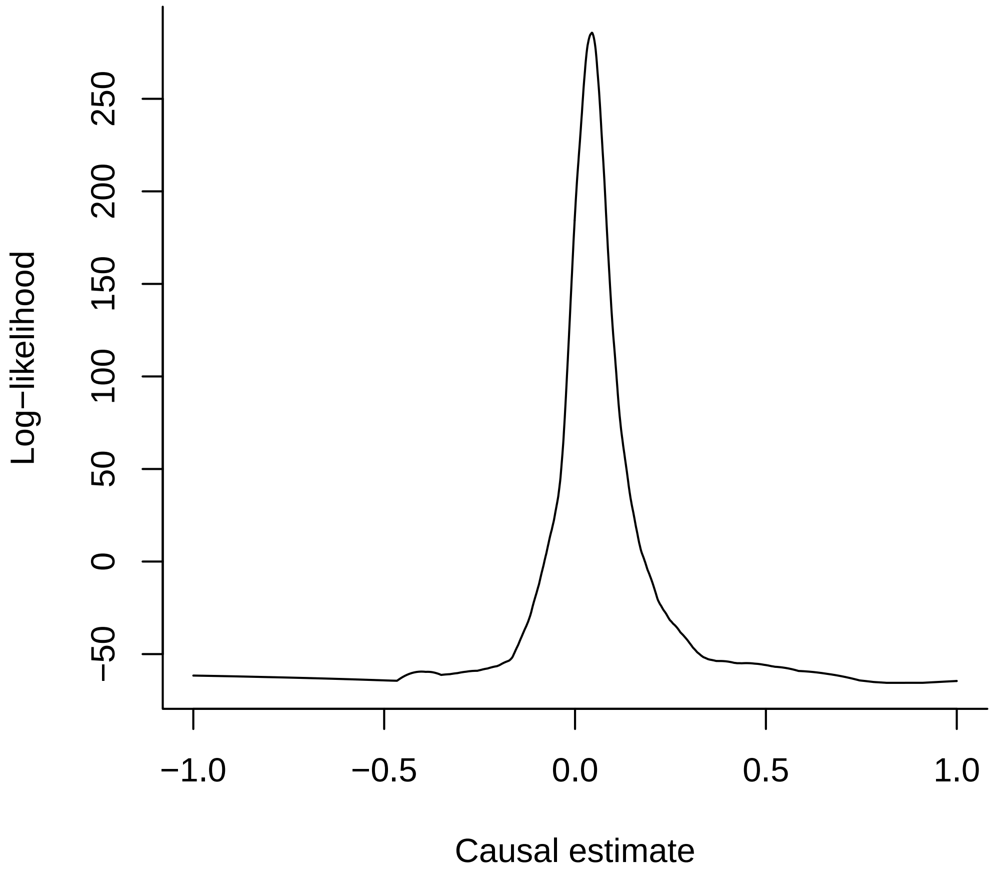

Figure A3: Log-likelihood from contamination mixture method as a function of causal estimate for applied example of systolic blood pressure and coronary artery disease risk.

Clustering results for additional applied example of HDL-cholesterol and coronary artery disease risk.

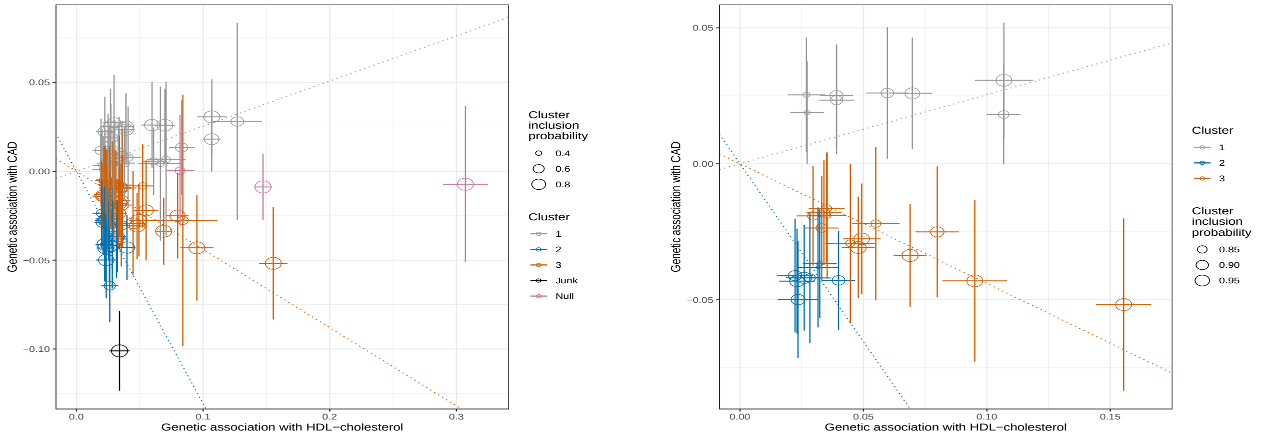

Figure A4: Clusters from genetic associations with HDL-cholesterol (mmol/L) and coronary artery disease risk (log odds) per HDL-cholesterol increase allele using MR-Clust. Left: method version (A) – each variant is assigned to the cluster with the greatest conditional probability. Right: method version (B) – variants are only assigned to a cluster if the conditional probability is ≥ 0.8, and clusters are only displayed if at least 4 variants are assigned to the cluster.
